# IDH1-mutant metabolite D-2-hydroxyglutarate inhibits proliferation and sensitizes glioma to temozolomide via down-regulating ITGB4/PI3K/AKT

**DOI:** 10.1038/s41420-024-02088-y

**Published:** 2024-07-09

**Authors:** Shuangmei Tong, Jian Wu, Yun Song, Wenhuan Fu, Yifan Yuan, Pin Zhong, Yinlong Liu, Bin Wang

**Affiliations:** 1 Department of Pharmacy, Huashan Hospital, Fudan University School of Medicine, Shanghai, 200040 China; 2 Department of Neurosurgery, Huashan Hospital, Fudan University School of Medicine, Shanghai, 200040 China

**Keywords:** Cancer therapy, CNS cancer

## Abstract

The heterogeneous molecular subtypes of gliomas demonstrate varied responses to chemotherapy and distinct prognostic outcomes. Gliomas with Isocitrate dehydrogenase 1 (IDH1) mutation are associated with better outcomes and are more responsive to temozolomide (TMZ) compared to those without IDH1 mutation. IDH1-mutant gliomas elevate D-2-hydroxyglutarate (D-2HG) levels, with potential dual effects on tumor progression. Limited research has explored the potential anti-glioma effects of D-2HG in combination with TMZ. Clinical data from over 2500 glioma patients in our study confirms that those with IDH1 mutations exhibit enhanced responsiveness to TMZ chemotherapy and a significantly better prognosis compared to IDH1 wild-type patients. In subsequent cellular experiments, we found that the IDH1-mutant metabolite D-2HG suppresses Integrin subunit beta 4 (ITGB4) expression, and down-regulate the phosphorylation levels of PI3K and AKT, ultimately inhibiting cell proliferation while promoting apoptosis, thereby improving glioma prognosis. Additionally, we have demonstrated the synergistic effect of D-2HG and TMZ in anti-glioma therapy involved inhibiting the proliferation of glioma cells and promoting apoptosis. Finally, by integrating data from the CGGA and TCGA databases, it was validated that ITGB4 expression was lower in IDH1-mutant gliomas, and patients with lower ITGB4 expression were associated with better prognosis. These findings indicate that ITGB4 may be a promising therapeutic target for gliomas and D-2HG inhibits proliferation and sensitizes glioma to temozolomide via down-regulating ITGB4/PI3K/AKT. These findings drive theoretical innovation and research progress in glioma therapy.

## Introduction

Glioma is a highly lethal central nervous system tumor characterized by a dismal prognosis [1], with a 90% recurrence rate, 80% of which manifest within 2 cm of the primary lesion [2, 3]. Current clinical approaches to glioma treatment primarily involve surgical interventions coupled with adjuvant radiation and chemotherapy [4]. Despite the emergence of innovative treatments such as targeted therapy, immunotherapy, and electric field therapy, their efficacy remains limited [5]. The most recent World Health Organization criteria have classified adult gliomas into two major categories: Isocitrate dehydrogenase 1 (IDH1) wild type and IDH1-mutant [6]. IDH1-mutant glioma patients typically exhibit enhanced responsiveness to temozolomide (TMZ) chemotherapy, along with significantly improved prognoses compared to those with IDH1 wild-type tumors [7, 8].

Although current evidence suggests that IDH1 mutation catalyzes the reduction of α-ketoglutarate (α-KG) into D-2-hydroxyglutarate (D-2HG) [9, 10], which has been linked to distinctive cancer metabolism and signaling cascade patterns, contributing to malignant transformation, progression, and therapeutic resistance [11–13]. However, clinical investigations have demonstrated that patients with IDH1 mutations experience significantly better clinical prognoses than those with wild-type gliomas (median overall survival: 3.8 years *vs*. 1.1 years) [7]. Furthermore, high levels of D-2HG (greater than 1.489 mM) have been associated with extended median overall survival (OS) in glioma patients (undetected *vs*. 2.2 years) [14]. It has also been established that D-2HG exerts anti-leukemia effects by regulating the FTO/m6A/MYC/CEBPA pathway and FTO/m6A/PFKP/LDHB axis [15, 16]. An experimental study indicated that IDH1 mutation and D-2HG could induce oxidative stress, autophagy, and apoptosis in glioma cells [17]. Based on these observations, we hypothesized that D-2HG itself may possess anti-glioma properties and could potentially enhance the anti-tumor effects of TMZ through a synergistic mechanism.

Herein, we conducted a retrospective clinical investigation to confirm that IDH1-mutant glioma patients indeed exhibited improved prognoses, greater responsiveness to TMZ treatment, and elevations of D-2HG in comparison to those with IDH1 wild-type gliomas. Subsequently, we study the potential anti-glioma effects of D-2HG in U251 glioma cells by assessing proliferation, migration, DNA damage, apoptosis, and using proteomics to explore the underlying mechanisms. Then we investigated whether a synergistic anti-glioma effect existed between D-2HG and TMZ to elucidate the mechanisms behind the improved efficacy of TMZ in IDH1-mutant glioma patients. Finally, we further verified the effects of D-2HG and TMZ in primary glioma cells and U251 cells stably expressing either IDH1*mut* or IDH1*wt*. This research endeavor contributes to a more comprehensive understanding of the role of D-2HG and ITGB4 in glioma and may facilitate the development of more effective therapeutic strategies.

## Results

### IDH1 mutation is an independent factor associated with a favorable prognosis and enhanced responsiveness to TMZ treatment in gliomas

**Fig. 1:**
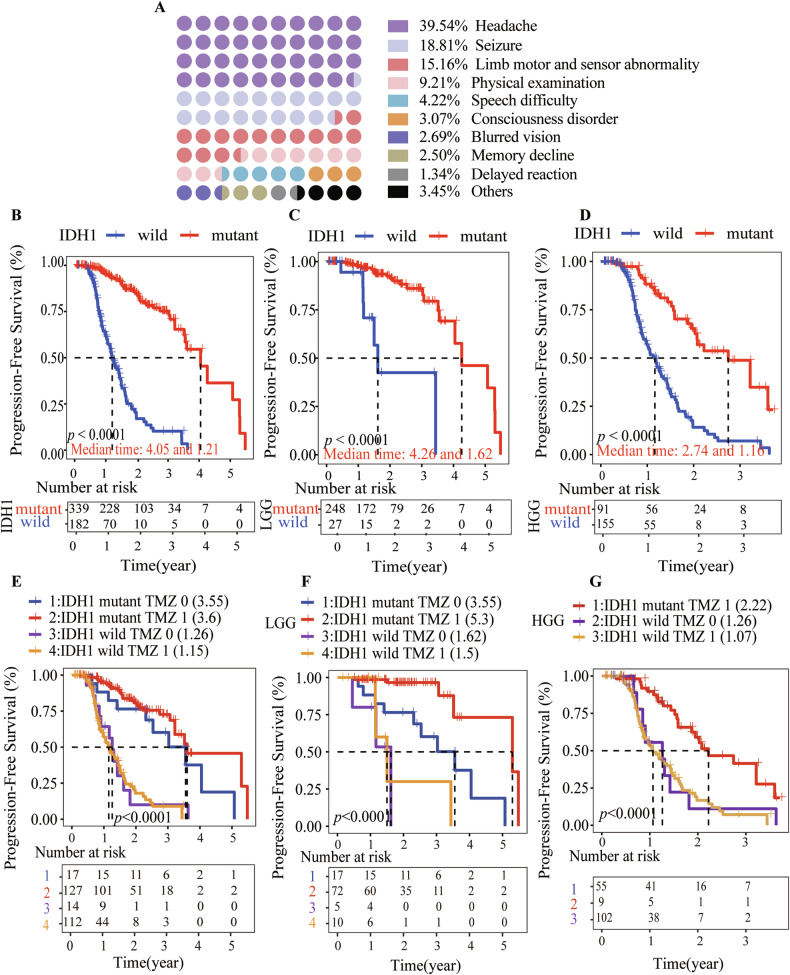
Clinical information of 521 glioma patients. **A** Initial Symptoms in Glioma Patients. Among 521 glioma patients, the most prevalent initial symptoms were dizziness and headache, accounting for 39.54%. Seizures were reported in 18.81% of cases, while limb movement and sensory abnormalities were observed in 15.16% of patients. **B** Among 521 cases of glioma, patients with IDH1 mutations had a longer progression-free survival (PFS) compared to those with wild-type IDH1, with a median PFS of 4.05 years compared to 1.21 years (*P* < 0.001). **C** Among 275 cases of low-grade gliomas (LGG), patients with IDH1 mutations had a median PFS of 4.26 years compared to 1.62 years for those with wild-type IDH1 (*P* < 0.001). **D** Among 246 cases of high-grade gliomas (HGG), patients with IDH1 mutations had a median PFS of 2.74 years compared to 1.16 years for those with wild-type IDH1 (*P* < 0.001). **E** Patients with IDH1-mutant gliomas had significantly longer progression-free survival (PFS) than those with IDH1 wild-type gliomas (*P* < 0.001), and TMZ treatment did not affect the PFS within IDH1-mutant and wild-type glioma groups. **F** In low-grade gliomas (LGG), TMZ extended the median PFS of patients with IDH1-mutant gliomas from 3.55 years to 5.3 years (*P* < 0.001), while it had no impact on the PFS of patients with IDH1 wild-type gliomas. **G** In high-grade gliomas (HGG), TMZ had no effect on the PFS of patients with IDH1 wild-type gliomas, and there were no cases of IDH1-mutant HGG that did not receive TMZ, so the treatment effect of TMZ on IDH1-mutant HGG was not analyzed.

We collected basic information from 182 IDH1 wild-type and 339 IDH1-mutant glioma patients in our hospital and analyzed various prognostic factors. Detailed clinical information about the patients is provided in Table 1. One-way ANOVA revealed that low-grade glioma, gross total resection, the presence of an IDH1 mutation, and tumors located in the frontal or temporal lobes were protective factors. Multivariate analysis further confirmed that tumor grade and the presence of an IDH1 mutation were independent prognostic factors (Table 1). And the most common initial symptoms of 521 glioma patients were dizziness and headache, followed by seizures, limb motor and sensory abnormalities (Fig. 1A).

**Table 1:** Patients characteristics and Cox analysis of prognostic factors of PFS in gliomas from our series.

Variables		Univariate analysis			Multivariate analysis		
	Number	HR	95% CI	*P*-value	HR	95% CI	*P*-value
Age							
<60	390		Reference				
≥60	131	2.9	2–4.1	<0.0001*	1.05	0.67–1.65	0.839086078
Sex							
Female	207		Reference				
Male	314	1.1	0.76–1.5	0.72	0.73	0.48–1.12	0.152117301
WHO class							
LGG	275						
HGG	246	7.8	5.1–12	<0.0001*	3.43	1.81–6.5	<0.0001*
IDH1 mutation							
No	182		Reference				
Yes	339	0.12	0.081–0.17	<0.0001*	0.25	0.15–0.43	<0.0001*
TMZ							
No	31		Reference				
Yes	239	0.83	0.52–1.3	0.45	0.58	0.3–1.12	0.10546914
Radiotherapy							
No	28		Reference				
Yes	208	1	0.62–1.7	0.89	1.53	0.8–2.94	0.201164703
Surgical resection							
STR	135	0.66	0.44–0.99	0.045*	1.03	0.41–2.6	0.94612077
GTR	365	1.2	0.81–1.7	0.38	1.02	0.45–2.32	0.96177589
Biopsy	21	3.2	1.6–6.3	<0.0001*			
Tumor location							
Bilateral	28	1.9	0.87–4	0.11	1.38	0.49–3.91	0.546238372
Left side	228	1	0.73–1.4	0.92			
Right side	265	0.9	0.65–1.2	0.53	1.35	0.88–2.07	0.164008474
Tumor site							
Parietooccipital	46	2.3	1.5–3.7	<0.0001*	0.88	0.36–2.14	0.783767334
Frontal temporal	416	0.59	0.4–0.86	0.0071*	0.91	0.41–2.02	0.818719069
Ventricle	4	3.00E-07	0-Inf	0.99	3.25E-08	0-Inf	0.994057817
Cerebellum	5	5.7	1.8–18	0.0032*	2.83	0.56–14.18	0.205841435
Other	50		Reference				

*LGG* low-grade glioma, *HGG* high-grade glioma, *STR* subtotal resection, *GTR* gross total resection.

*Statistically significant difference (*P* < 0.05).

Subsequently, we confirmed that IDH1-mutant gliomas demonstrated a more favorable outcome than IDH1 wild type in our cohort, 916 cases from the Chinese Glioma Genome Atlas (CGGA) and 868 cases from The Cancer Genome Atlas (TCGA) databases (detailed information is provided in Figs. 1B–D and S1A–F). We next investigated the effect of IDH1 mutation on the efficacy of TMZ in these patients based on PFS and OS: patients with IDH1 mutation had a more significant response to TMZ than those with IDH1 wild type (Figs. 1E–G, S1G–L).

And it is noteworthy that even without TMZ treatment, patients with IDH1-mutant gliomas exhibit better therapeutic efficacy compared to those with wild-type tumors who receive TMZ. We hypothesize that D-2HG produced by IDH1 mutation may have anti-cancer properties in gliomas, leading to better prognosis in IDH1-mutant cases. We then assess D-2HG levels and its anti-glioma efficacy to determine the differences between IDH1-mutant and wild-type gliomas.

### IDH1-mutant metabolite D-2-hydroxyglutarate showed anti-glioma effect

**Fig. 2:**
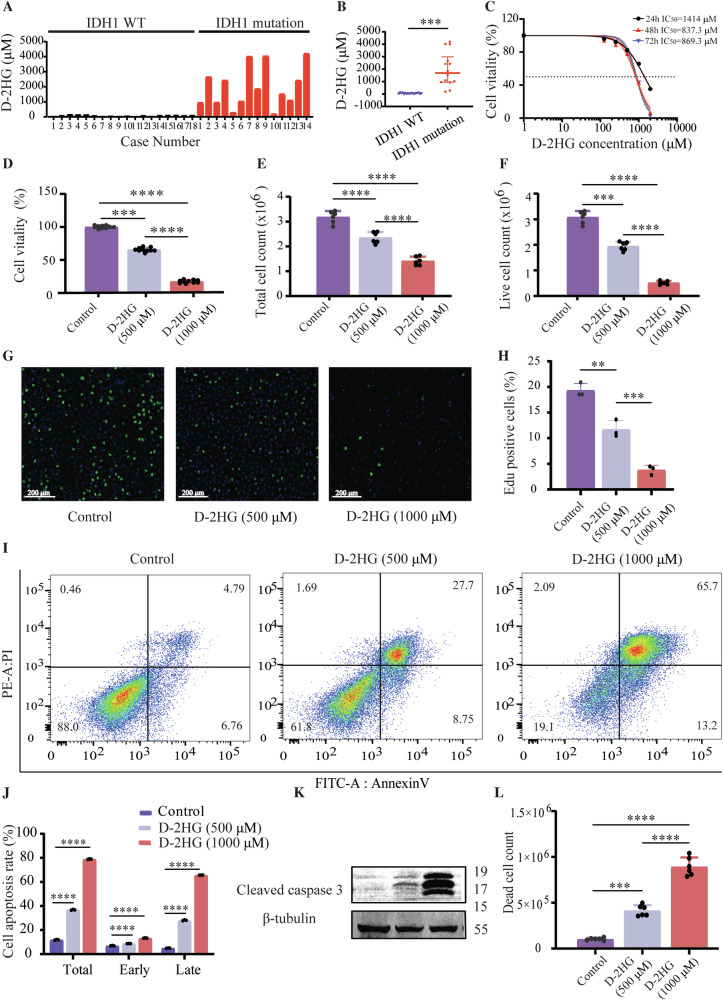
IDH1-mutant metabolite D-2-hydroxyglutarate showed anti-glioma effect. **A** The Concentration of D-2HG in 14 IDH1-Mutant and 18 IDH1 Wild-Type Glioma Tissues. **B** The average concentration of D-2HG in IDH1 Wild-Type gliomas was 70.9 μM, whereas the average D-2HG concentration in IDH1-mutant gliomas was 1.9 mM, showing a statistically significant difference, *P* < 0.001. **C** IC_50_ values of D-2HG in U251 cells at 24 h, 48 h, and 72 h were 1414 μM, 837.3 μM, and 869.3 μM, respectively. IC_50_ of D-2HG was determined using the “log(inhibitor) vs. Normalized response -Variable slope” method in GraphPad Prism 9. **D** Cell viability. **E** Reduce total cell count. **F** The number of viable cells. **G** EdU assay was conducted to evaluate the DNA replication ability. **H** Quantification of EdU assay. **I** Cell apoptosis detected by flow cytometry. **J** Quantification of Cell apoptosis. **K** The cleaved caspase-3 expression. **L** Death cell counts.

We tested D-2HG levels in 14 IDH1-mutant and 18 IDH1 wild-type glioma tissues using HPLC-MS/MS. Results showed significantly higher D-2HG levels in IDH1-mutant gliomas (1.9 mM) compared to wild-type gliomas (70.9 μM) (Fig. 2A, B). Supplementary Table 1 and Supplementary 1 provide detailed information about the 32 patients in this analysis.

And the next part of our study was focused on exploring the potential anti-glioma effects of D-2HG and its underlying mechanisms. We treated glioma U251 cells with varying concentrations of D-2HG for 24, 48, and 72 h. The IC_50_ values of D-2HG in U251 cells were 1414 μM, 837.3 μM, and 869.3 μM at 24, 48, and 72 h, respectively (Fig. 2C). The most significant inhibitory effect on glioma cell activity was observed at 48 h after a single dose of D-2HG.

Subsequently, U251 cells were treated with 500 μM and 1000 μM of D-2HG for 48 h, resulting in decreased cell viability (Fig. 2D), total cell count (Fig. 2E), viable cell count (Fig. 2F), and DNA replication (Fig. 2G, H). Additionally, D-2HG induced significant apoptosis in U251 cells (Fig. 2I), particularly late-stage apoptosis (Fig. 2J), as indicated by high levels of cleaved caspase-3 (Fig. 2K). This led to a substantial amount of cell death in the cells (Fig. 2L).

### D-2HG downregulated ITGB4/PI3K/AKT in the glioma cell line

**Fig. 3:**
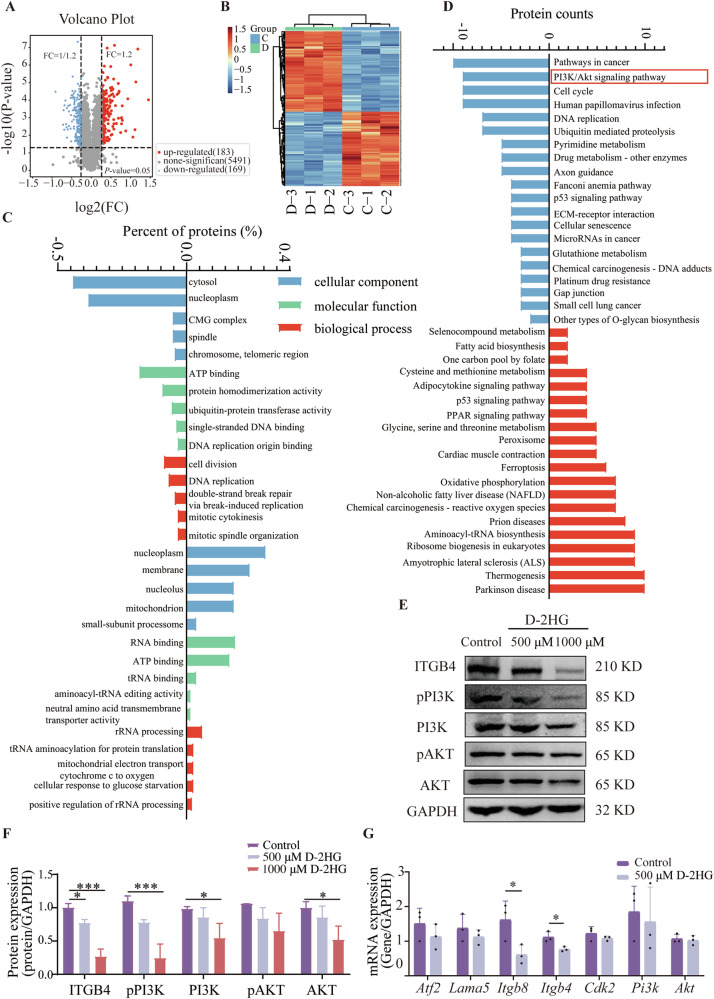
Proteomic analysis to identify the potential mechanism of D-2HG. **A** The volcano plot depicts red dots representing upregulated proteins, blue dots indicating downregulated proteins, and gray dots representing proteins with non-significant differential expression. **B** The heat maps of differentially expressed proteins at different treatment groups, with red denoting high expression and blue denoting low expression. Each row represents the expression of each protein in different groups. **C** GO enrichment analysis of differential protein. The horizontal axis represents the names of GO terms, while the vertical axis represents the percentage of differential proteins annotated to that entry. Negative values indicate downregulation and positive values indicate upregulation. **D** KEGG analysis indicated the signaling pathways affected by D-2HG. The horizontal axis represents the names of KEGG pathways, while the vertical axis represents the number of differential proteins annotated to that pathway. Negative values (blue) indicate downregulation, and positive values (red) indicate upregulation. **E**, **F** Western blot analysis of protein levels. **G** qPCR analysis of RNA transcription level.

To explore the anti-glioma mechanism of D-2HG, we analyzed the effects of D-2HG on U251 cells by conducting a proteomic analysis. We found 352 proteins that were differentially expressed, with 183 upregulated and 169 downregulated proteins (Fig. 3A). Cluster analysis showed significant differences between the treated and control groups (Fig. 3B). Gene Ontology (GO) enrichment analysis of the 352 differential proteins indicated that downregulated proteins were primarily in the cytoplasm and nucleoplasm, impacting ATP binding and protein homodimerization. Upregulated proteins were primarily in the cytoplasm and cell membrane, influencing RNA binding and ATP binding, and impacting processes related to rRNA and tRNA metabolism (Fig. 3C).

KEGG pathway analysis revealed that downregulated proteins in the D-2HG treatment group were mainly involved in tumor-related pathways, PI3K/AKT pathway, and cell cycle, while upregulated proteins were associated with Parkinson’s disease, thermogenesis, and amyotrophic lateral sclerosis pathways compared to the control group (Fig. 3D). Coincidentally, the PI3K/AKT pathway was overexpressed in glioma tissues, which could enhance glioma cell proliferation and migration [18]. Several studies have provided evidence that the inhibition of the PI3K/AKT pathway effectively suppresses the growth of gliomas [19]. And ITGB4 is upstream of PI3K/AKT (as evidenced by the KEGG website), and the relationship between ITGB4 and the PI3K/AKT pathway has also been reported in renal cancer and colorectal cancer [20, 21]. Therefore, our subsequent research will focus on the ITGB4/PI3K/AKT pathway.

To confirm our results, U251 cells were exposed to different concentrations of D-2HG and analyzed using western blot and qPCR. D-2HG inhibited ITGB4 (*P* < 0.001) expression and decreased PI3K (*P* < 0.001) and pPI3K levels with a stronger effect at higher concentrations, and 1000 μM D-2HG can down-regulate the expression of AKT (*P* = 0.009) while having no significant effect on pAKT (Fig. 3E, F). qPCR showed significant differences in *Itgb4* (*P* = 0.02) and *Itgb8* (*P* = 0.04) compared to the control group (Fig. 3G).

### ITGB4 downregulation is related to a lower proliferation of glioma cells and better prognosis for glioma patients

**Fig. 4:**
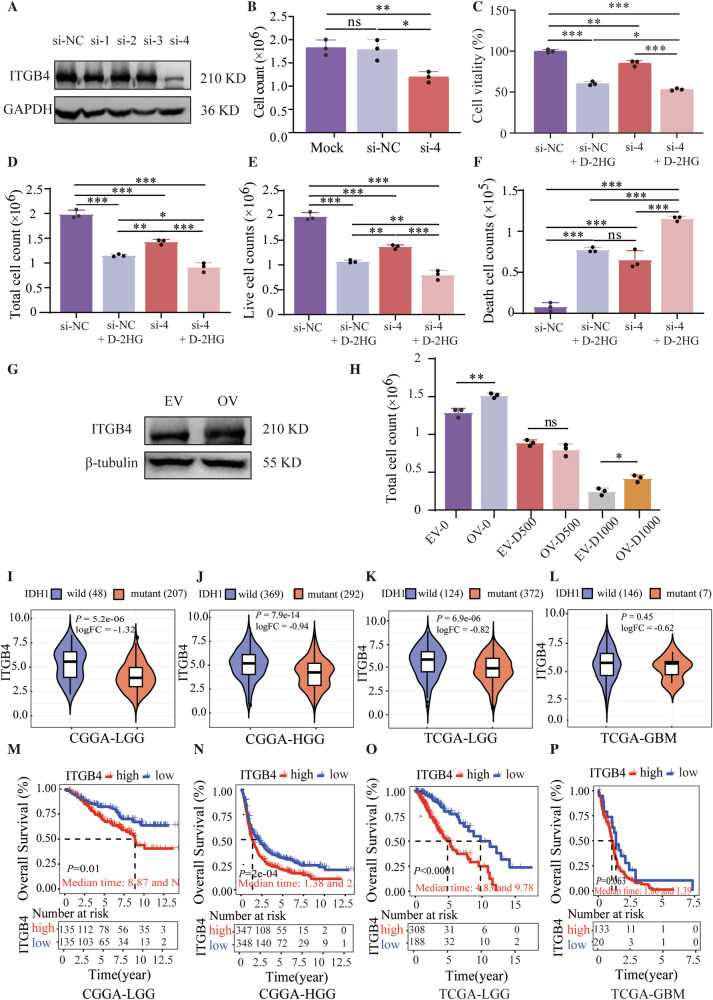
ITGB4 downregulation is related to a lower proliferation of glioma cells and better prognosis for glioma patients. **A** ITGB4 protein expression in *Itgb4* siRNA-transfected cells. **B** Cell proliferation was measured by Trypan blue staining. **C** Same number of ITGB4 siRNA-transfected and siNC-transfected cells were treated with D-2HG for 48 h. Cell viability was measured by CCK-8 assay. **D** Total cell count, **E** Live cell counts, **F** Dead cell counts were measured by Trypan blue staining. **G** ITGB4 protein expression in ITGB4-overexpressed cells. **H** ITGB4-overexpressed enhances the proliferation of glioma cells while simultaneously weakening the anti-tumor effect of D-2HG. **I**–**L** The relationship between ITGB4 expression and IDH1 mutation, the numbers in brackets represent the number of cases. **M**–**P** The relationship between ITGB4 expression and the prognosis of glioma patients.

**Fig. 5:**
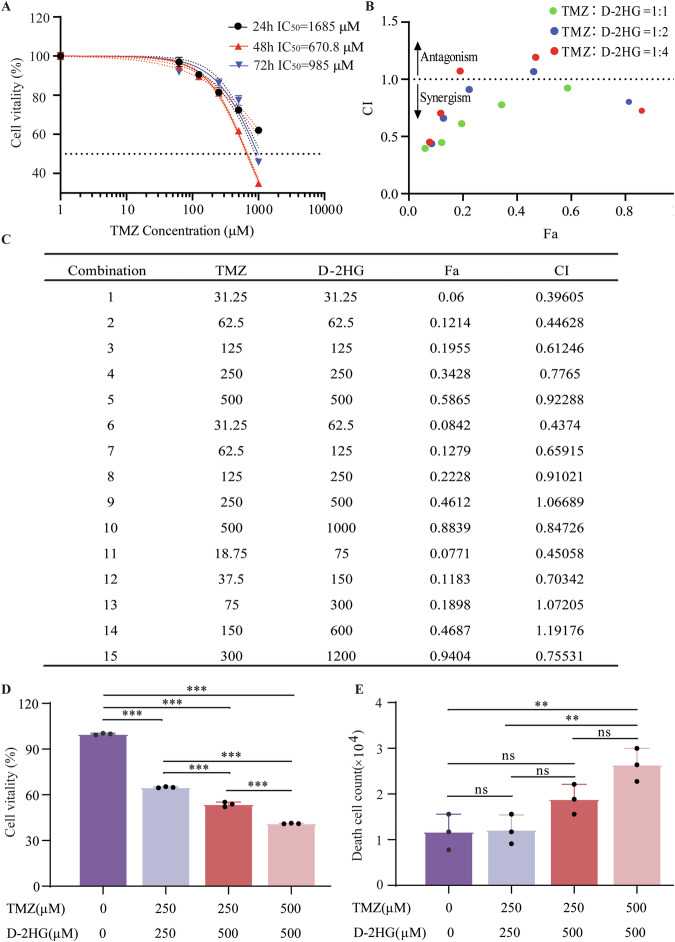
Combination of D-2HG and TMZ triggers synergistic anti-glioma activity. **A** IC_50_ curves of TMZ in U251 cells at 24 h, 48 h, and 72 h, respectively. **B**, **C** Isobologram analysis shows the synergistic cytotoxic effect of D-2HG plus TMZ. CI < 1 indicates synergy. **D** Cell viability and **E** Cell toxicity of U251 treated with four combinations of D-2HG and TMZ.

To explore the biological functions of *Itgb4* in glioma and treatment, we established cell lines with altered *Itgb4* levels and assessed their proliferation capacity and sensitivity to D-2HG. Out of 4 siRNAs tested, only si-4 successfully reduced *Itgb4* levels (Figs. 4A and S2A, B). U251 cells transfected with si-4 showed decreased proliferation (Fig. 4B, *P* = 0.0073) and viability (Fig. S2C, *P* < 0.001) compared to the control group after 3 days. *Itgb4* knockdown cells had lower viability (Fig. 4C, *P* < 0.001), reduced proliferation (Figs. 4D and 5E, *P* < 0.001), and a higher number of dead cells (Fig. 4F, *P* < 0.001) after D-2HG treatment. In contrast, *Itgb4*-overexpressing cells (Figs. 4G and S2D) displayed increased cell proliferation and diminished sensitivity to D-2HG (Fig. 4H), indicating that the downregulation of *Itgb4* may contribute to the toxicity of D-2HG.

To further explore the role of *Itgb4* in gliomas, we analyzed the relationship between *Itgb4* expression and IDH1 status in both CGGA and TCGA databases. *Itgb4* expression was lower in IDH1-mutant gliomas compared to IDH1 wild-type gliomas in both LGG and HGG (Fig. 4I–K, *P* < 0.001), except for GBM in TCGA where there was only a slight decrease with no significant difference (Fig. 4L, *P* = 0.45, logFC = −0.62).

Furthermore, we examined the relationship between *Itgb4* expression and the prognosis of glioma patients. In CGGA and TCGA, low *Itgb4* expression was associated with significantly longer OS than high expression. In CGGA, the median OS of LGG was not reached for the low *Itgb4* expression group versus 8.87 years for the high expression group (Fig. 4M, *P* = 0.01), as for HGG, the median OS was 2.13 years for low *Itgb4* expression versus 1.38 years for high expression (Fig. 4N, *P* < 0.001). In TCGA, the median OS for LGG with high *Itgb4* expression was 4.83 years, compared to 9.78 years for low *Itgb4* expression (Fig. 4O, *P* < 0.001). However, there was no significant difference in median OS between patients with high and low *Itgb4* expression in GBM (1.06 years vs. 1.39 years, *P* = 0.063) (Fig. 4P).

### D-2HG and temozolomide exert synergistic effects on glioma cell line

Given that D-2HG reportedly possesses a promising anti-glioma effect, we hypothesized that a synergistic relationship might exist between D-2HG and TMZ, rendering IDH1-mutant glioma patients more sensitive to TMZ treatment. We first explored the time/dose toxicity of TMZ in U251 cells. The IC_50_ values of TMZ in U251 cells at 24, 48, and 72 h were 1685 μM, 670.8 μM, and 985 μM, respectively (Fig. 5A). These findings indicated that the optimal inhibition of TMZ in U251 cells occurs at 48 h, which coincides with the duration of action of D-2HG (Fig. 2C). Next, we applied Chou-Talalay isobologram analysis to evaluate potential synergistic interactions. U251 cell lines were treated with various concentrations of D-2HG and TMZ for 48 h. A combination index (CI) of less than 1 suggested a synergistic effect. The results showed that most combination CI values were less than 1 (Fig. 5B, C), implying that the two drugs act synergistically at the modified concentration. This phenomenon suggests that the combination of D-2HG and TMZ might explain why IDH1-mutant patients exhibit enhanced sensitivity to TMZ treatment with the combination showing therapeutic potential in glioma treatment.

We tested four combinations of D-2HG and TMZ for cell activity and toxicity, finding that 500 μM D-2HG combined with 500 μM TMZ had the best anti-glioma cell activity and promoted cell death (Fig. 5D, E). Subsequent functional experiments were conducted using this modified concentration combination.

### D-2HG works synergistically with TMZ to inhibit glioma cell proliferation and migration

**Fig. 6:**
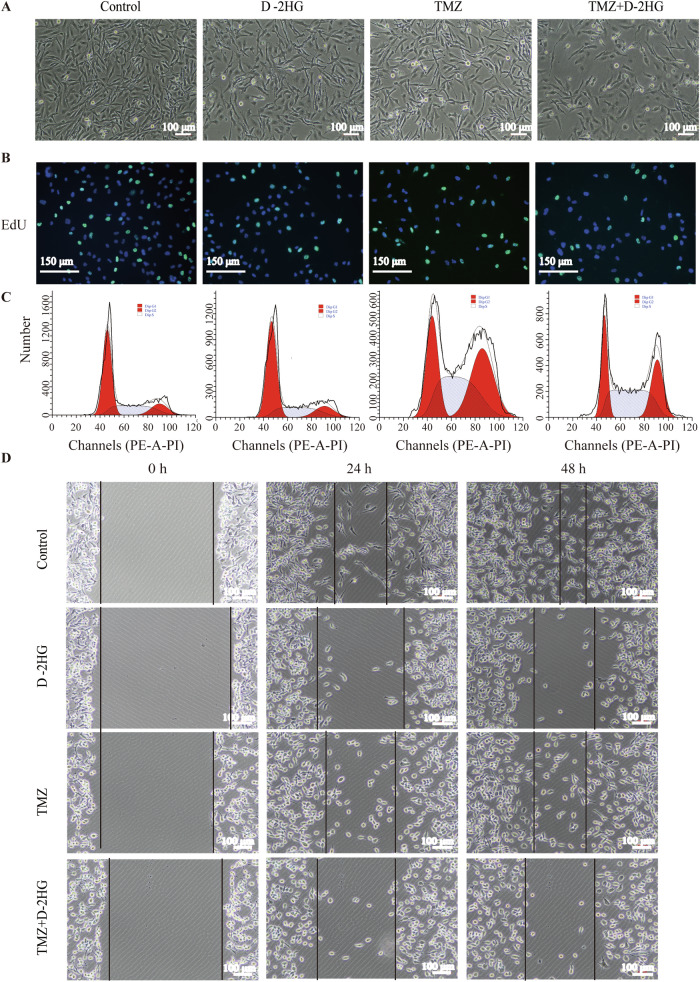
D-2HG enhances the anti-proliferative effect of TMZ. U251 cells were treated with D-2HG (500 μM), TMZ (500 μM), or a combination of the two drugs. Representative images of U251 proliferation (**A**), DNA replication ability (**B**), and cell migration (**D**) taken by a microscope. **C** Cell cycle detected via flow cytometry.

Treating U251 cells with 500 μM D-2HG, 500 μM TMZ, and 500 μM D-2HG + 500 μM TMZ for 48 h resulted in decreased cell abundance compared to the control group, with the combination treatment showing the most significant reduction (Figs. 6A and S3A). Besides, a proliferation EdU assay showed that the combination of D-2HG and TMZ inhibited cell proliferation more significantly than either D-2HG or TMZ alone (Fig. 6B). Quantification of the EdU assay is shown in Fig. S3B. In addition, we observed that D-2HG increased G1 phase cells while decreasing G2 phase cells. In contrast, TMZ and TMZ + D-2HG decreased G1 phase cells and increased S and G2 phases. Notably, the combination of D-2HG and TMZ significantly increased S phase cells compared to individual D-2HG or TMZ treatments (Figs. 6C and S3C). Furthermore, scratch assays revealed that the control group demonstrated the highest migratory ability, with migration rates of 48% at 24 h and 72% at 48 h. D-2HG effectively inhibited cell migration, with migration rates of 20% at 24 h and 35% at 48 h. In comparison, the migration rates at 24 and 48 h after TMZ treatment were 35% and 52%, respectively. When D-2HG and TMZ were used in combination, cell migration was significantly inhibited, reaching 11% at 24 h and 17% at 48 h (Figs. 6D and S3D). The study showed that combining D-2HG and TMZ was more effective in inhibiting glioma cell proliferation and migration compared to either drug alone, suggesting that combination therapy is more effective against glioma.

### D-2HG and IDH1 mutation enhance TMZ-induced apoptosis through down-regulating ITGB4/PI3K/AKT pathway

**Fig. 7:**
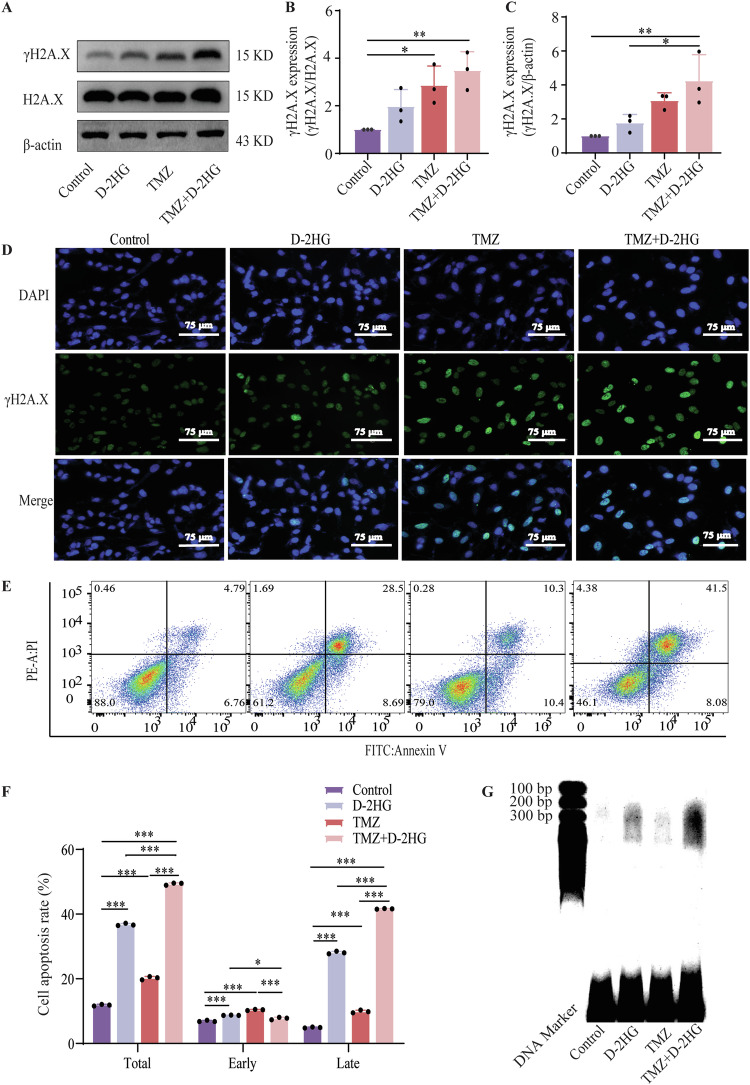
D-2HG enhances the DNA damage and apoptosis induced by TMZ. **A**–**C** Western blotting measuring the expression of γH2A.X in U251 and glioma primary cells induced by D-2HG (500 μM), TMZ (500 μM), and a combination of the two drugs. **D** Immunofluorescent staining of γH2A.X in U251 and glioma primary cells with normal (control) and treatment groups. **E** Representative images of cell apoptosis detected through flow cytometry after a 48-h treatment. **F** Percentages of early-stage and late-stage apoptotic and their sum were calculated and compared among groups. **G** The combination of D-2HG and TMZ induces extensive DNA breakage and cell death, confirmed by DNA gel electrophoresis.

Subsequently, we investigated whether D-2HG could enhance the cytotoxic effects of TMZ. TMZ induces DNA damage, primarily double-strand breaks and apoptosis. In consideration of γH2A.X as a biomarker for DNA double-strand breaks, we assessed the γH2A.X levels via western blotting and immunofluorescence. Western blotting showed that the combined administration of D-2HG and TMZ moderately increased the expression of γH2A.X; however, this increase was not statistically significant (Fig. 7A–C). Immunofluorescence of γH2A.X showed increased DNA damage in the D-2HG + TMZ group (*P* < 0.05, Figs. 7D and S4A). Flow cytometry revealed all drugs induced apoptosis, with D-2HG + TMZ significantly increasing late apoptosis (Fig. 7E, F, *P* < 0.001). After drug treatment, agarose gel electrophoresis showed indistinct and dispersed bands within the low molecular weight range, indicating DNA fragmentation and necrosis, particularly in the D-2HG + TMZ group (Fig. 7G).

**Fig. 8:**
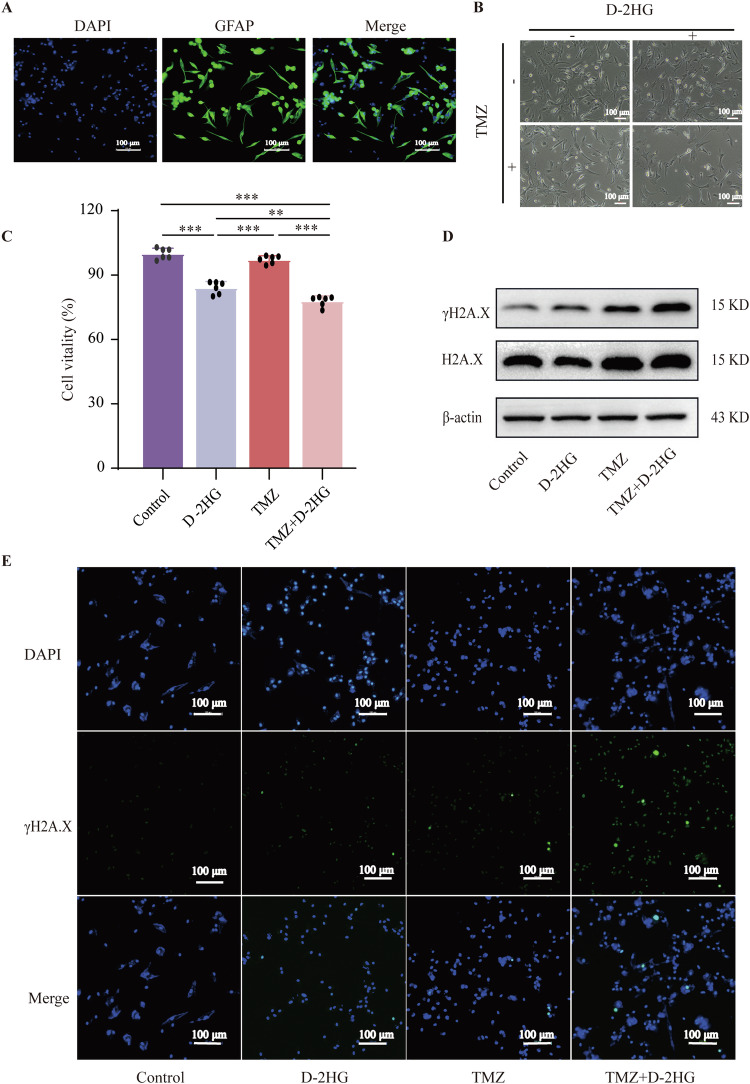
The effect of D-2HG combined with TMZ in primary glioma cells. **A** Primary glioma cells verified by GFAP. D-2HG exhibits a significant inhibitory effect on the proliferation (**B**) and cell viability (**C**) of glioma primary cells and enhances the DNA damage of TMZ (**D**, **E**).

In parallel, we conducted a study on the combined treatment of D-2HG and TMZ in primary glioma cells (Fig. 8A). The results demonstrated that the combination of D-2HG and TMZ effectively suppressed the proliferation (Fig. 8B) and viability (Fig. 8C) of primary glioma cells, while concurrently increasing DNA damage (Figs. 8D–E and S4B).

**Fig. 9:**
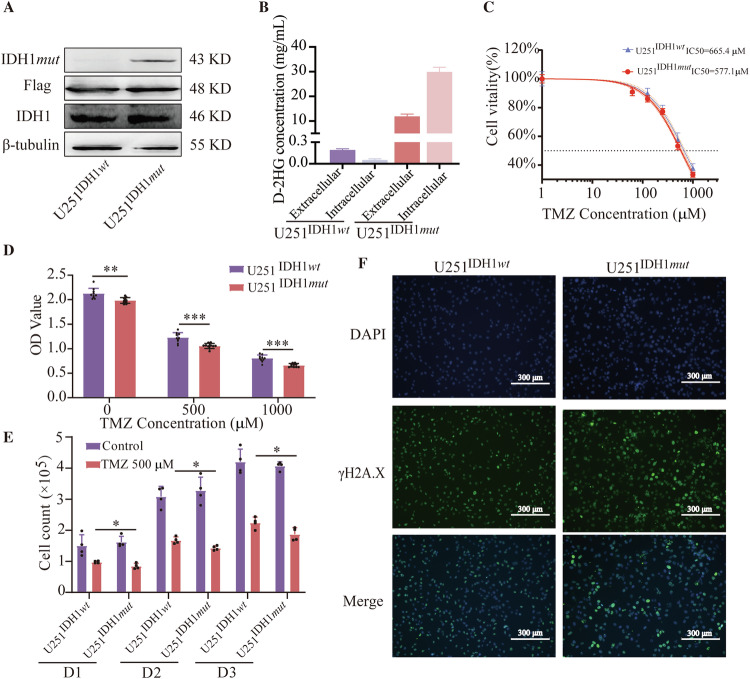
IDH1 mutation and D-2HG sensitized glioma cells to TMZ. **A**, **B** Western blot and HPLC/MS results indicate that we successfully generated U251 cell lines with overexpressed IDH1 mutation. The intracellular concentration of D-2HG in U251^IDH1*mut*^ was the highest, reaching 29.7 mg/mL (approximately 200.7 μM). The supernatant of U251^IDH1*mut*^ contained 11.5 mg/mL (approximately 77.7 μM) of D-2HG. In contrast, both intracellular and supernatant D-2HG concentrations in U251^IDH1*wt*^ cells remained notably low. **C** U251^IDH1*mut*^ cells are more sensitive to TMZ, with an IC_50_ value of 577.1 μM, which is lower than the IC_50_ value of U251^IDH1*wt*^ cells (665.4 μM). TMZ treatment induces **D** Lower cell viability, **E** Lower cell proliferation capabilities, and **F** Higher expression of γH2A.X and more severe DNA damage in U251^IDH1*mut*^ cells, compared to U251^IDH1*wt*^ cells.

The preceding research demonstrates the potent anti-glioma efficacy of exogenously introduced D-2HG in conjunction with TMZ. Consequently, we established stable IDH1*mut* and IDH1*wt*-expressing cell lines to corroborate the enhancing effect of endogenous D-2HG on TMZ, cell lines confirmed by IDH1-mutant protein level (Fig. 9A) and the concentration of D-2HG (Fig. 9B). The toxicity of TMZ demonstrated that U251^IDH1*mut*^ cells exhibited increased sensitivity to TMZ, with an IC_50_ value of 577.1 μM, while the IC_50_ value for U251^IDH1*wt*^ cells was 665.4 μM (Fig. 9C). This was also confirmed by the lower viability (Fig. 9D) and cell proliferation (Fig. 9E) of U251^IDH1*mut*^ cells treated with TMZ compared with U251^IDH1*wt*^ cells. Immunofluorescence results indicated that U251^IDH1*mut*^ cells displayed more intense γH2A.X foci than U251^IDH1*wt*^ cells after TMZ treatment (Figs. 9F and S4C). These results suggest that U251^IDH1*mut*^ cells capable of producing endogenous D-2HG exhibit increased sensitivity to TMZ.

**Fig. 10:**
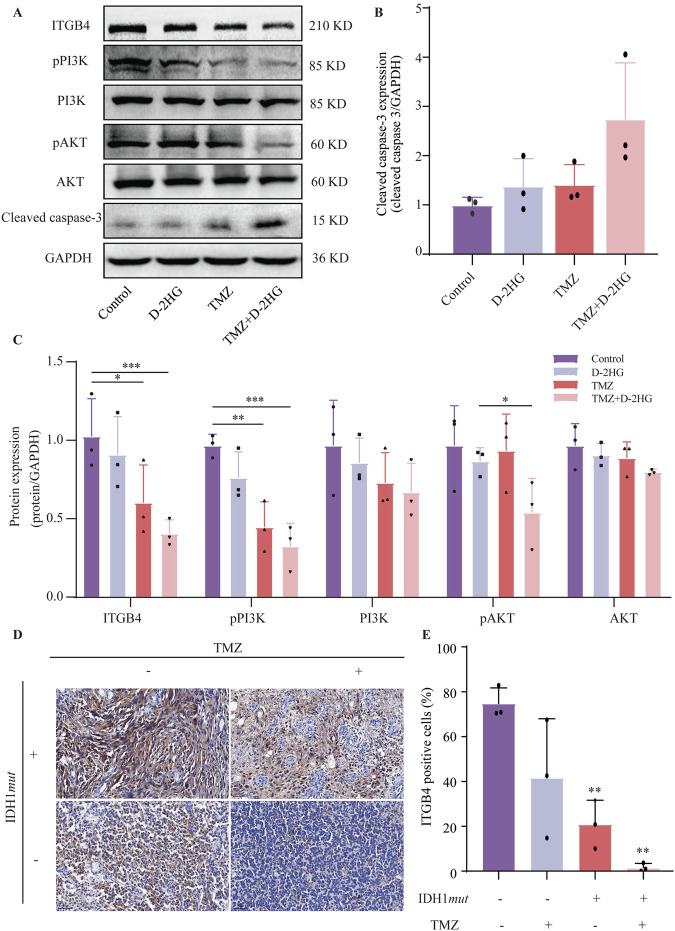
D-2HG in combination with TMZ inhibits the ITGB4/PI3K/AKT pathway and elevates the level of apoptosis in glioma U251 cells. **A**–**C** Western blotting measuring the expression of key molecules in the ITGB4/PI3K/AKT pathway in U251 cells treated with D-2HG (500 μM), TMZ (500 μM), or a combination of the two drugs. **D**, **E** The expression level of ITGB4 in clinical glioma tissues was validated through immunohistochemistry.

Finally, we evaluated the effects of D-2HG combined with TMZ on the ITGB4/PI3K/AKT pathway and cell apoptosis. Cleaved caspase-3 levels increased (Fig. 10A, B), while ITGB4 expression and PI3K/AKT phosphorylation decreased (Fig. 10A, C) in the treated group compared to the control group. These findings support the idea that D-2HG combined with TMZ inhibits the ITGB4/PI3K/AKT pathway. To confirm the connection between ITGB4, D-2HG, and TMZ, glioma tissues were grouped by IDH1 status and TMZ use (primary or recurrent glioma). Results showed that IDH1 wild-type gliomas without TMZ had the highest ITGB4 expression, while IDH1-mutant gliomas with TMZ had the lowest (Fig. 10D, E). This suggests that IDH1 mutation and TMZ treatment decrease ITGB4 expression in gliomas.

## Discussion

**Fig. 11:**
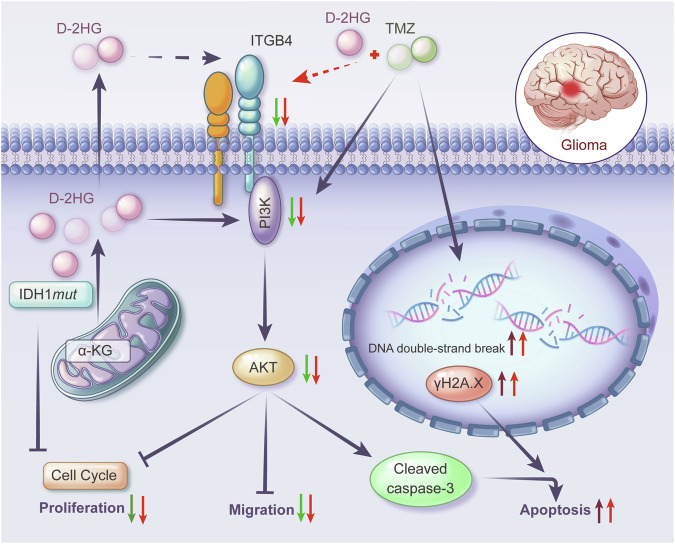
Schematic model for the synergism of IDH1-mutant metabolite D-2-hydroxyglutarate and temozolomide in anti-glioma via down-regulating ITGB4/PI3K/AKT and increasing DNA double-strand break.

Our results present evidence supporting a significantly better prognosis, TMZ treatment efficacy, and higher D-2HG level in IDH1-mutant gliomas compared to their IDH1 wild-type counterparts (Figs. 1, S1, and 2). Subsequently, we introduced unprecedented evidence demonstrating that D-2HG inhibits glioma cell proliferation and enhances apoptosis by down-regulating the ITGB4/PI3K/AKT pathway (Fig. 11). Moreover, our external database validation revealed lower *Itgb4* expression in IDH1-mutant gliomas, correlating with a more favorable prognosis. We further confirmed the synergistic relationship between D-2HG and TMZ, which includes the inhibition of proliferation, migration, an increase in DNA damage, and a subsequent exacerbation of apoptosis. Additionally, we confirmed that U251 cells with stable IDH1 mutation were more sensitive to TMZ.

D-2HG has been identified as an oncometabolite linked to tumorigenesis due to its ability to cause genomic and epigenomic dysregulation and inhibit cell differentiation, as previously reported [10]. However, some scholars have also suggested that D-2HG may possess anti-leukemic and anti-glioma effects [15–17]. As summarized in a review by Huang et al. [22], whether IDH1 mutation is a ‘friend or foe’ largely depends on the specific model system being used. Compared with normal brain tissue, IDH1 mutations, and D-2HG have been identified as driving factors in tumor development and targets for specific therapy in IDH1-mutant glioma models. Numerous clinical trials of the IDH1-mutant inhibitor Ivosidenib are currently underway [23]. However, when compared to IDH1 wild-type gliomas, IDH1 mutations and high levels of D-2HG are markers of better prognosis and are associated with anti-tumor growth effects [24]. These contrasting conclusions lead us to speculate that IDH1-mutant tumor cells have developed a tolerance to IDH1 mutation and D-2HG, enabling their continued survival. In contrast, IDH1 wild-type tumors are responsive to D-2HG. Consequently, the specific mechanisms of D-2HG are intricate and require further elucidation.

In our experimental model, D-2HG demonstrated potential anti-glioma effects by inhibiting the expression of Integrin subunit beta 4 (ITGB4), which subsequently downregulated the phosphorylation levels of PI3K and AKT, ultimately suppressing glioma cell proliferation and promoting apoptosis. The PI3K/AKT pathway is important in glioma, with studies showing over-expression of key molecules in glioma tissues that promote cell proliferation and migration. Some scholars have investigated the anti-glioma effect induced by inhibiting the PI3K/AKT pathway [19]. ITGB4, belonging to the integrin protein family located on the cell membrane, plays a pivotal role in cell adhesion, migration, proliferation, and signal transduction [25]. Interestingly, ITGB4 can activate the PI3K/AKT pathway, further promoting the survival, proliferation, and migration of tumor cells. Current research has demonstrated that ITGB4 is a critical molecule in maintaining glioma stemness and promoting glioma growth [26]. Thus, inhibiting ITGB4 reduces the self-renewal ability, thereby hindering glioma development [26]. Our experimental data shows that D-2HG can reduce ITGB4 expression. Bioinformatics analysis suggests that *Itgb4* expression is lower in IDH1-mutant gliomas, and lower *Itgb4* levels are associated with better prognosis. However, the mechanism behind D-2HG’s effect on ITGB4 expression warrants further study.

Glioma, as an intracranial malignant tumor, presents a challenging prognosis, limited therapeutic options, and a postoperative recurrence rate of up to 90% [3]. Further research on clinical treatment is necessary to create better treatment strategies. Combining therapies has shown to be more effective in clinical practice [27]. Our study demonstrates that D-2HG in collaboration with TMZ, exerts a more potent anti-glioma effect, which provides an experimental basis for exploring novel combination therapies for gliomas. However, further clarification is warranted regarding animal experiments involving the interaction of D-2HG and TMZ. Moreover, additional efforts should be undertaken to evaluate the potential of D-2HG in treating glioma patients, including a more comprehensive investigation of its ability to penetrate the blood-brain barrier.

Collectively, our results shed light on the potential new strategies for glioma treatment. And our research also suggests that ITGB4 may serve as a potential therapeutic target for gliomas, and D-2HG may exert anti-glioma effects by inhibiting ITGB4.

## Conclusions

In summary, our study, drawing from clinical data of over 2500 glioma patients, confirms the superior prognosis of IDH1-mutant glioma patients compared to those with IDH1 wild type, coupled with their heightened sensitivity to TMZ. Cell experiments for the first time reveal that the IDH1 mutation byproduct, D-2HG, effectively inhibits the ITGB4/PI3K/AKT pathway, thus exerting potent anti-glioma effects and offering insights into its mechanism of action, including its synergistic effect with TMZ. Furthermore, our research offers plausible molecular explanations for the improved prognosis of IDH1 mutation-type gliomas and their enhanced responsiveness to TMZ. Moreover, our findings suggest ITGB4 as a promising therapeutic target for gliomas and lay the groundwork for the translational development of D-2HG as a potential anti-glioma therapeutic agent. Ongoing investigations involving glioma xenograft models are currently underway to further assess the in vivo anti-glioma efficacy.

## Methods

### Patient cohort

The inclusion criteria for this retrospective study were as follows: (1). Patients diagnosed with histologically confirmed glioma at Huashan Hospital, Fudan University, from January 2011 to December 2021, with no concurrent diseases, and who had not undergone any anti-tumor treatment prior to surgery. (2). Patients with complete clinical data, including gender, age, presenting symptoms, tumor pathology results, tumor location, tumor size, and extent of surgical resection. (3). Pathological results clearly indicating IDH1 negativity or positivity (i.e., IDH1 mutation type or wild type). (4). Age range: 18–80 years old. Exclusion criteria were: (1). Incomplete clinical information. (2). Patients in the pregnant state. (3). Patients participating in other clinical trials during the same period. Through further screening during the study, a total of 521 cases with valid data were obtained.

Database cohorts: one sourced from the TCGA (The Cancer Genome Atlas) database, which comprised 868 patients with available follow-up data, and the other from the CGGA (Chinese Glioma Genome Atlas) database, consisting of a total of 965 glioma patients with available follow-up information.

### Clinical glioma samples

Glioma tissues were collected from the Neurological Surgery Department of Huashan Hospital between January 2021 and December 2022. Glioma tissues were obtained during surgical resection, fresh samples were used for the detection of D-2HG level, protein expression, and primary glioma cell culture. Consent forms were obtained from all patients. The collection of human glioma specimens was approved by the local ethics committee in Huashan Hospital, Fudan University (No. 2020-1185).

### D-2HG quantification

Tissues D-2HG level were measured by using high-performance liquid chromatography-electrospray tandem mass spectrometry (HPLC-MS/MS) as previously [28]. D-2HG (Yuanye) as a standard sample for its quantification.

### Cell line and cell culture

The human glioma cell line U251 was purchased from FuHeng Biology and regularly confirmed via STR typing. These cells were cultured in Dulbecco’s Modified Eagle Medium/Nutrient Mixture F-12 (DMEM/F-12, Gibco, USA) supplemented with 10% (v/v) fetal bovine serum (Gibco). All cells were maintained in a humidified atmosphere containing 5% CO_2_ at 37 °C.

### CCK-8 assay

Cell viability was measured by CCK-8 kit (CCK-8, Dojindo) following the manufacturer’s protocol. Cells were seeded at 5000 cells/well in 96-well plates. After 24 h of incubation, cells were treated with DMSO or a range of doses of temozolomide (MCE), (2 R)-Octyl-α-hydroxyglutarate ((2 R)-Octyl-2-HG, MCE), and their combination. After 48 h, 10 μL CCK-8 was added to each well. The absorbance (A) at 450 nm was measured using the Tecan spark spectrophotometer (Tecan Group Ltd., Zurich, Switzerland) after another 1.5 h of incubation at 37 °C. Cell survival rate was calculated as follows: cell survival rate (%) = [(A_drug group_ − A_blank group_)/(A_control group_ − A_blank group_)] × 100%. TMZ and D-2HG concentrations that achieved 50% growth inhibition (IC_50_) were calculated from cell survival curves using GraphPad Prism.

### Trypan blue viability assay

Trypan blue was used for live/dead staining and cell count. After treatment, cells trypsinized and stained with Trypan blue solution (0.4%, Biosharp) and then photographed, 3 fields were randomly selected to calculate the mean cell number. All cell counts were made using a CountStar IC1000 analysis system (Ruiyu, Shanghai, China).

### EdU assay

The proliferation of cells was performed using an EdU kit (Beyotime). Cells were planted in Chamber Slide (Lab-Tek) and incubated with different concentrations of drugs for 48 h. Then, 2× EdU working solution was added for 2 h at 37 °C, followed by fixation with 4% paraformaldehyde for 20 min and permeabilization with 0.1% TritonX-100 (Solarbio) for 15 min. Then, Click Reaction Mixture and Hoechst were used to stain cells. Imaging was performed with Invitrogen EVOS M5000 microscopes. Positive cells were analyzed with Image J software.

### Cell cycle arrest assay

Cell cycle assay was performed to evaluate the effects of TMZ and D-2HG on U251 cell cycle progression. U251 cells were seeded at 1.5 × 10^5^ cells per well on 6-well plates and incubated overnight. Then, cells were treated with vehicle, TMZ (500 μM), D-2HG (500 μM), or both drugs in combination (500 μM + 500 μM) for 48 h. The treated cells from the well were collected and fixed with cold ethanol at −20 °C for 24 h, treated with RNase, stained with a cell cycle kit for 30 min in darkness, and analyzed by a flow cytometer (BD).

### Apoptosis assay

Annexin V-FITC/propidium iodide apoptosis kit (BD) was used to detect cell apoptosis as follows. U251 cells were seeded in 6-well plates and cultured for 24 h. After 48 h of drug treatment, the cells were harvested and resuspended in a 1× binding buffer containing FITC-conjugated Annexin V and PI in the dark. Then, stained samples were analyzed by flow cytometry (BD). The experiments were repeated at least three times.

### Wound healing assay

For the wound healing assay to assess cell migration, U251 cells were grown in 6-well plates for 24 h to 95% confluency. Scratch-wounds were made on the well using 200 μL sterile pipette tips. Then, the cells were washed twice with PBS to remove the floating cells. Cell culture media containing concentrations of drugs were added to the petri dishes. Photographs were captured at specified time intervals for assessment.

### RNA extraction, reverse transcription, and quantitative real-time PCR assays

Total RNA in the cultured cells was extracted using TRIZOL reagent (Invitrogen) according to the manufacturer’s protocol. RNA quantity and quality were evaluated on a NANO Quant infinite M200Pro (TECAN) and RNA electrophoresis. A total of 2 μg of RNA was used to obtain complementary DNA by reverse transcription using an Invitrogen Reverse Transcription Kit. qPCR reactions were done with 2× PCR MIX (Qiagen) using an ABI ViiA 7 real-time quantitative PCR system. Experiments were run in triplicate. The comparative threshold cycle (ΔΔCT) was used to calculate the mRNA level of the target gene. The mean of internal reference genes, GAPDH, was used to normalize the expression of each transcript. All primers were designed using Primer Express version 3.0 (Applied Biosystems) and synthesized by HuaDa gene. The corresponding primers used during qPCR are listed in Supplementary Table 2.

### Western blot assay

At the end of treatment, the cells were washed twice with ice-cold phosphate-buffered saline (PBS). The total protein was extracted in RIPA cell lysis buffer (Beyotime) containing 1 × PI, 1 × PPIA, 1 × PPIB, and 1 mM PMSF for 30 min on ice. Protein quantification was performed using a BCA protein assay kit (Thermo Fisher Scientific, Waltham, MA, USA). Aliquots of each sample were loaded and separated on 8–12% SDS-PAGE gel electrophoresis, then transferred to a PVDF membrane at 300 mA for 30–240 min. After blocking the membranes for one hour at room temperature with 5% non-fat milk or 3% bovine serum albumin (BSA) in 1/TBST, the specific primary antibodies were incubated at 4 °C overnight. The membrane was washed and incubated with a secondary antibody for 2 h at room temperature after primary antibody incubation. ECL solution was used to detect membranes after incubation. ImageJ was used to perform the protein quantification. Cleaved caspase-3, pPI3K (Tyr458), PI3K, pAKT (Ser473), AKT, GAPDH, β-actin, and β-tubulin (1:1000, Cell Signaling Technology, MA), ITGB4 (1:1000, Abcam) were used as primary antibodies. Full and uncropped western blots can be found in Supplemental Material.

### DNA ladder

DNA fragmentation was detected using a DNA ladder extraction kit (Beyotime) according to the manufacturer’s protocol. DNA extraction was quantified by a spectrophotometer (NanoDrop 2000, Wilmington, USA) and separated on 1% agarose gels and UV visualization using a Bio-Rad GelDoc XR System (Bio-Rad Laboratories, Inc.) after staining with Gel Red (Invitrogen).

### Immunofluorescence assay

Immunofluorescence assay was used to detect γH2A.X expression in cells. After different treatments, the cells were cultured on glass coverslips in 12-well plates and fixed with 4% paraformaldehyde for 15 min at room temperature. The cells were then permeabilized with PBS with 1% Trixon-100 and 1% FBS for 15 min, and blocked with PBS with 0.1% Trixon-100 and 10% FBS for 2 h at room temperature and then incubated with the primary antibody (1:200, Abmart) overnight at 4 °C. After that, the cells were incubated at room temperature for 60 min with FITC-tagged secondary antibodies (1:1000, Abcam). Following that, cells were washed, and DAPI (Beyotime Company, China) was used to counterstain nuclei. Invitrogen EVOS M5000 microscopes were used for imaging.

### Immunohistochemistry assay

ITGB4 (1:200, Abcam, Germany) antibodies were used for immunohistochemical staining as it was described previously [29]. ITGB4 expression under a light microscope was observed. The optical density values of each group were analyzed and measured using Image J software.

### Lentivirus infection

The lentiviral expression vectors carrying the cDNA for *Itgb4*, IDH1*mut*, and IDH1*wt*, as well as a control lentiviral, were obtained from Jikai Co. (Shanghai, China) and were used to infect U251 cells. Subsequently, A stable cell line overexpressing *Itgb4*, IDH1*mut*, IDH1*wt*, or an empty vector was developed by puromycin selection and used for in vitro experiments.

### siRNA transfection

U251 cells were transfected with siRNA reagents using HiPerFect Transfection Reagent (Qiagen) according to the manufacturer’s protocol. Small interfering RNA (siRNA) that was against ITGB4 (si-*Itgb4*) mRNA was produced by Gene Pharma (Gene Pharma) as previously described [30], siRNA targeting *Itgb4* are as follows: si-1: CAGAAGAUGUGGAUGAGUU, si-2: CAGGAAGATTCATCCAACA, si-3: GGUCACCUCCAAGAUGUUC, si-4: GGAAAGAGCTGCAGGTGAA. RNA interference (siNC) was used as a negative control. After transfection for 72 h, the gene silencing activities were assessed by quantitative real-time polymerase chain reaction (qRT-PCR) and western blot (WB) measurements.

### Tandem mass tag (TMT)-based Proteomics

A TMT-based proteomic study was conducted to detect differences in protein expression between groups, as previously described [31]. U251 cells were treated with either DMSO or 500 μM D-2HG for 48 h, and total proteins in the samples were extracted. These proteins underwent trypsin enzymolysis and TMT labeling, followed by mixing for chromatographic separation. Subsequently, the samples were analyzed by LC–MS/MS using an Easy-nLC 1200 system (Thermo Scientific). GO and KEGG pathway analyses were employed to identify the most significantly differentially expressed proteins. Proteins exhibiting an expression fold change of ≥1.2 or a fold change of ≤1/1.2, with a *P*-value of <0.05, were filtered as differentially expressed proteins between groups.

### Statistical analysis

Patient PFS was calculated from the date of primary surgery to the date of recurrence or the end of the study, while OS was calculated from the date of primary surgery to the date of death or the end of the study. The prognostic value of IDH mutation and TMZ treatment in glioma patients was evaluated using Log-rank survival curves, and prognostic factors were assessed using the Cox proportional hazards model with the R survival package. Unpaired Student’s *t*-test was used for comparison between the two groups, and a one-way ANOVA and the Turkey test were used to compare differences among groups. *P*-values < 0.05 were statistically significant.

### Supplementary information

Supplementary figs and tables

Supplementary 1. Chromatographic profiles of D-2HG from the glioma tissues.

Supplemental Material-original western blots

## Data Availability

The mass spectrometry proteomics data have been deposited to the ProteomeXchange Consortium (https://proteomecentral.proteomexchange.org) via the iProX partner repository [32, 33] with the dataset identifier PXD052264. Any other data are available upon reasonable request to the corresponding author.

## References

[1] Li T, Li J, Chen Z, Zhang S, Li S, Wageh S (2022). Glioma diagnosis and therapy: current challenges and nanomaterial-based solutions. J Control Release.

[2] Stupp R, Hegi ME, Mason WP, van den Bent MJ, Taphoorn MJ, Janzer RC (2009). Effects of radiotherapy with concomitant and adjuvant temozolomide versus radiotherapy alone on survival in glioblastoma in a randomised phase III study: 5-year analysis of the EORTC-NCIC trial. Lancet Oncol.

[3] Lili Q, Haibin S, Liliang S (2022). Short term and long-term efficacy of bevacizumab monoclonal antibody and apatinib combined with temozolomide in treatment of recurrent high-grade glioma. China J Mod Med.

[4] Tong S, Wang Y, Wu J, Long J, Zhong P, Wang B (2021). Comprehensive pharmacogenomics characterization of temozolomide response in gliomas. Eur J Pharm.

[5] Nabors LB, Portnow J, Ahluwalia M, Baehring J, Brem H, Brem S (2020). Central nervous system cancers, version 3.2020, NCCN clinical practice guidelines in oncology. J Natl Compr Cancer Netw.

[6] Berger TR, Wen PY, Lang-Orsini M, Chukwueke UN (2022). World Health Organization 2021 classification of central nervous system tumors and implications for therapy for adult-type gliomas: a review. JAMA Oncol.

[7] Parsons DW, Jones S, Zhang X, Lin JC, Leary RJ, Angenendt P (2008). An integrated genomic analysis of human glioblastoma multiforme. Science.

[8] SongTao Q, Lei Y, Si G, YanQing D, HuiXia H, XueLin Z (2012). IDH mutations predict longer survival and response to temozolomide in secondary glioblastoma. Cancer Sci.

[9] Dang L, White DW, Gross S, Bennett BD, Bittinger MA, Driggers EM (2009). Cancer-associated IDH1 mutations produce 2-hydroxyglutarate. Nature.

[10] Reiter-Brennan C, Semmler L, Klein A (2018). The effects of 2-hydroxyglutarate on the tumorigenesis of gliomas. Contemp Oncol.

[11] Waitkus MS, Diplas BH, Yan H (2018). Biological role and therapeutic potential of IDH mutations in cancer. Cancer Cell.

[12] Chou FJ, Liu Y, Lang FC, Yang CZ (2021). D-2-hydroxyglutarate in glioma biology. Cells-Basel.

[13] Notarangelo G, Spinelli JB, Perez EM, Baker GJ, Kurmi K, Elia I (2022). Oncometabolite d-2HG alters T cell metabolism to impair CD8(+) T cell function. Science.

[14] Natsumeda M, Igarashi H, Nomura T, Ogura R, Tsukamoto Y, Kobayashi T (2014). Accumulation of 2-hydroxyglutarate in gliomas correlates with survival: a study by 3.0-tesla magnetic resonance spectroscopy. Acta Neuropathol Commun.

[15] Su R, Dong L, Li CY, Nachtergaele S, Wunderlich M, Qing Y (2018). R-2HG exhibits anti-tumor activity by targeting FTO/m (6)A/MYC/CEBPA signaling. Cell.

[16] Qing Y, Dong L, Gao L, Li CY, Li YC, Han L (2021). R-2-hydroxyglutarate attenuates aerobic glycolysis in leukemia by targeting the FTO/m (6)A/PFKP/LDHB axis. Mol Cell.

[17] Gilbert MR, Liu Y, Neltner J, Pu H, Morris A, Sunkara M (2014). Autophagy and oxidative stress in gliomas with IDH1 mutations. Acta Neuropathol.

[18] Khabibov M, Garifullin A, Boumber Y, Khaddour K, Fernandez M, Khamitov F (2022). Signaling pathways and therapeutic approaches in glioblastoma multiforme (Review). Int J Oncol.

[19] Barzegar Behrooz A, Talaie Z, Jusheghani F, Los MJ, Klonisch T, Ghavami S (2022). Wnt and PI3K/Akt/mTOR survival pathways as therapeutic targets in glioblastoma. Int J Mol Sci.

[20] Liu Z, Sun T, Piao C, Zhang Z, Kong C (2022). METTL14-mediated N6-methyladenosine modification of ITGB4 mRNA inhibits metastasis of clear cell renal cell carcinoma. Cell Commun Signal.

[21] Thongchot S, Singsuksawat E, Sumransub N, Pongpaibul A, Trakarnsanga A, Thuwajit P (2020). Periostin regulates autophagy through integrin α5β1 or α6β4 and an AKT-dependent pathway in colorectal cancer cell migration. J Cell Mol Med.

[22] Huang LE (2019). Friend or foe-IDH1 mutations in glioma 10 years on. Carcinogenesis.

[23] Abou-Alfa GK, Macarulla T, Javle MM, Kelley RK, Lubner SJ, Adeva J (2020). Ivosidenib in IDH1-mutant, chemotherapy-refractory cholangiocarcinoma (ClarIDHy): a multicentre, randomised, double-blind, placebo-controlled, phase 3 study. Lancet Oncol.

[24] Sim HW, Nejad R, Zhang W, Nassiri F, Mason W, Aldape KD (2019). Tissue 2-hydroxyglutarate as a biomarker for isocitrate dehydrogenase mutations in gliomas. Clin Cancer Res.

[25] Genduso S, Freytag V, Schetler D, Kirchner L, Schiecke A, Maar H (2023). Tumor cell integrin β4 and tumor stroma E-/P-selectin cooperatively regulate tumor growth in vivo. J Hematol Oncol.

[26] Ma B, Zhang L, Zou Y, He R, Wu Q, Han C (2019). Reciprocal regulation of integrin beta4 and KLF4 promotes gliomagenesis through maintaining cancer stem cell traits. J Exp Clin Cancer Res.

[27] National Health Commission of the People’s Republic of China. (2019). Diagnosis and treatment specification of glioma in children (2021 edition). J Multidiscip Cancer Manag.

[28] Cheng QY, Xiong J, Huang W, Ma Q, Ci W, Feng YQ (2015). Sensitive determination of onco-metabolites of D- and L-2-hydroxyglutarate enantiomers by chiral derivatization combined with liquid chromatography/mass spectrometry analysis. Sci Rep.

[29] Meng X, Liu P, Wu Y, Liu X, Huang Y, Yu B (2020). Integrin beta 4 (ITGB4) and its tyrosine-1510 phosphorylation promote pancreatic tumorigenesis and regulate the MEK1-ERK1/2 signaling pathway. Bosn J Basic Med Sci.

[30] Liu C, Xiang Y, Liu H, Li Y, Tan Y, Zhu X (2010). Integrin beta4 was downregulated on the airway epithelia of asthma patients. Acta Biochim Biophys Sin.

[31] Li DF, Cui ZH, Wang LY, Zhang KH, Cao LT, Zheng SJ (2021). Tandem mass tag (TMT)-based proteomic analysis of Cryptosporidium andersoni oocysts before and after excystation. Parasit Vectors.

[32] Ma J, Chen T, Wu S, Yang C, Bai M, Shu K (2019). iProX: an integrated proteome resource. Nucleic Acids Res.

[33] Chen T, Ma J, Liu Y, Chen Z, Xiao N, Lu Y (2022). iProX in 2021: connecting proteomics data sharing with big data. Nucleic Acids Res.

