# Supplementary material for: IDH1-mutant metabolite D-2-hydroxyglutarate inhibits proliferation and sensitizes glioma to temozolomide via down-regulating ITGB4/PI3K/AKT
Source: Cell Death Discov. 2024 Jul 9;10:317. doi: 10.1038/s41420-024-02088-y (PMC11233597; doi:10.1038/s41420-024-02088-y)
Supplement: Supplementary file 1 — Supplementary figs and tables [file 41420_2024_2088_MOESM1_ESM.doc]

**IDH1-mutant metabolite D-2-Hydroxyglutarate inhibits proliferation and sensitizes glioma to Temozolomide via down-regulating ITGB4/PI3K/AKT.**

**Supplementary Figures**


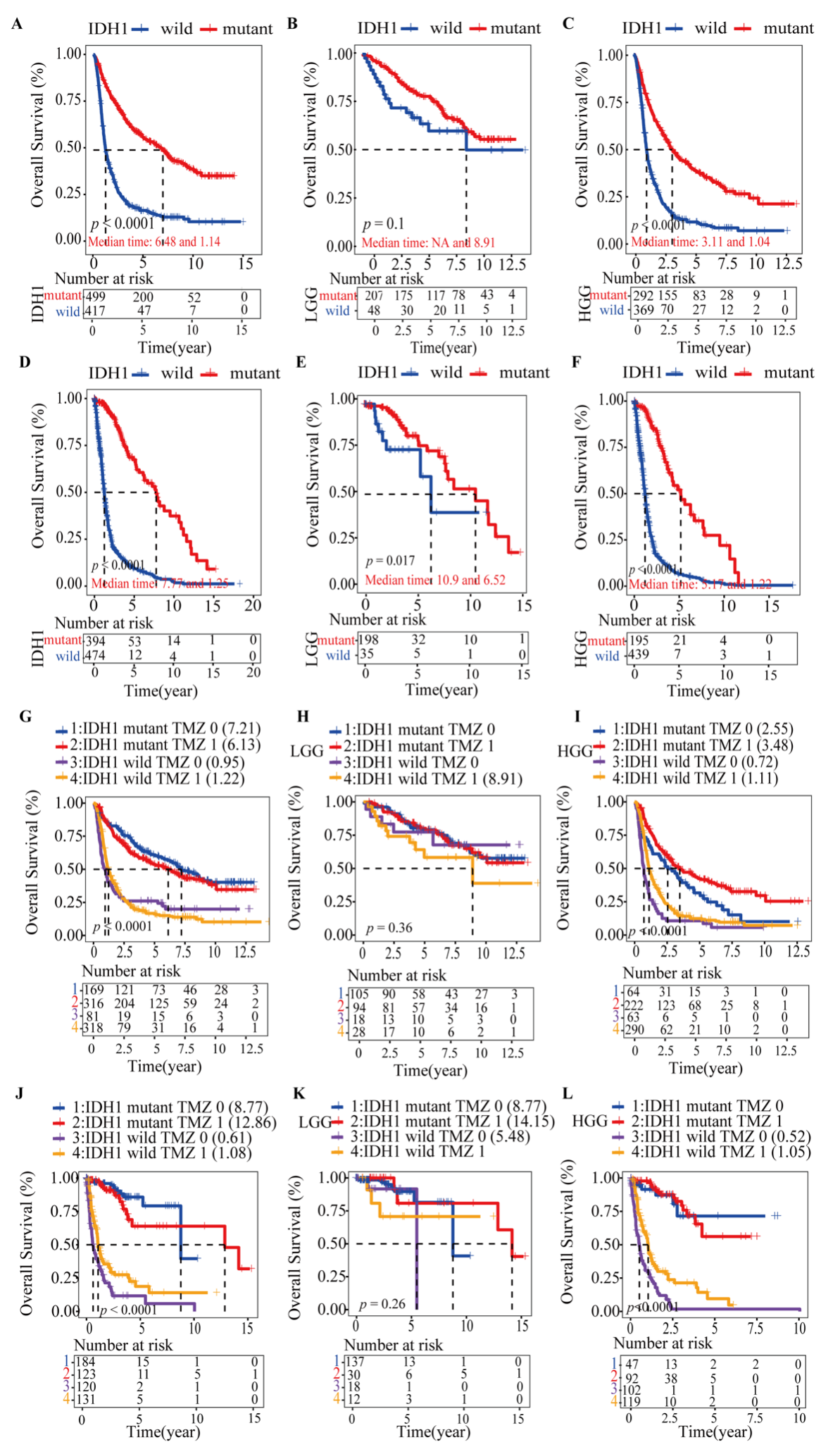


Supplementary Figure 1. The Impact of IDH1 Mutation on Prognosis and the Efficacy of Temozolomide. In CGGA database, (A) Among 916 cases of glioma, patients with IDH1 mutations had a longer overall survival (OS) compared to those with wild-type IDH1, with a median OS of 6.48 years compared to 1.14 years (*P*<0.001); (B) Among 255 cases of low-grade gliomas (LGG), patients with IDH1 mutations and wild-type IDH1 had a median OS of undetected and 8.91 years, respectively (*P*=0.1); (C) Among 661 cases of high-grade gliomas (HGG), patients with IDH1 mutations and wild-type IDH1 had a median OS of 3.11 years and 1.04 years, respectively (*P*<0.001). In TCGA, (D) Among 868 cases of glioma, patients with IDH1 mutations had a longer overall survival (OS) compared to those with wild-type IDH1, with a median OS of 7.77 years compared to 1.25 years (*P*<0.001); (E) Among 233 cases of LGG, patients with IDH1 mutations and wild-type IDH1 had a median OS of 10.9 years and 6.52 years, respectively (*P*=0.045); (F) Among 634 cases of HGG, patients with IDH1 mutations and wild-type IDH1 had a median OS of 5.17 years and 1.22 years, respectively (*P*<0.001). In CGGA, (G) Patients with IDH1 mutant gliomas had significantly longer overall survival (OS) than those with IDH1 wild-type gliomas (*P*<0.001), and whether patients received TMZ treatment had no impact on the overall OS within IDH1 mutant and wild-type glioma groups; (H) In LGG, TMZ had no impact on the overall OS of patients with both IDH1 mutant and wild-type gliomas; (I) In HGG, TMZ improved patient OS: the median OS of IDH1 mutant HGG patients increased from 2.55 years to 3.48 years after receiving TMZ (*P*<0.001), and the median OS of IDH1 wild-type HGG patients increased from 0.72 years to 1.11 years after receiving TMZ (*P*<0.001). In TCGA, (J) IDH1 mutation and TMZ treatment can prolong the overall survival (OS) of glioma patients. IDH1 mutant patients who received TMZ had their median OS extended from 8.77 years to 12.86 years (*P*<0.001), while IDH1 wild-type patients who received TMZ saw their median OS increase from 0.61 years to 1.08 years; (K) In LGG, IDH1 mutant patients who received TMZ had their median OS extended from 8.77 years to 14.15 years, but due to an insufficient number of samples for IDH1 wild-type patients who received TMZ, the median OS was undetermined, and further studies could not be conducted; (L) In HGG, TMZ significantly improved the prognosis of IDH1 wild-type patients, with their median OS increasing from 0.52 years to 1.05 years (*P*<0.001). However, among IDH1 mutant patients, the median OS was undetermined due to limited follow-up time.


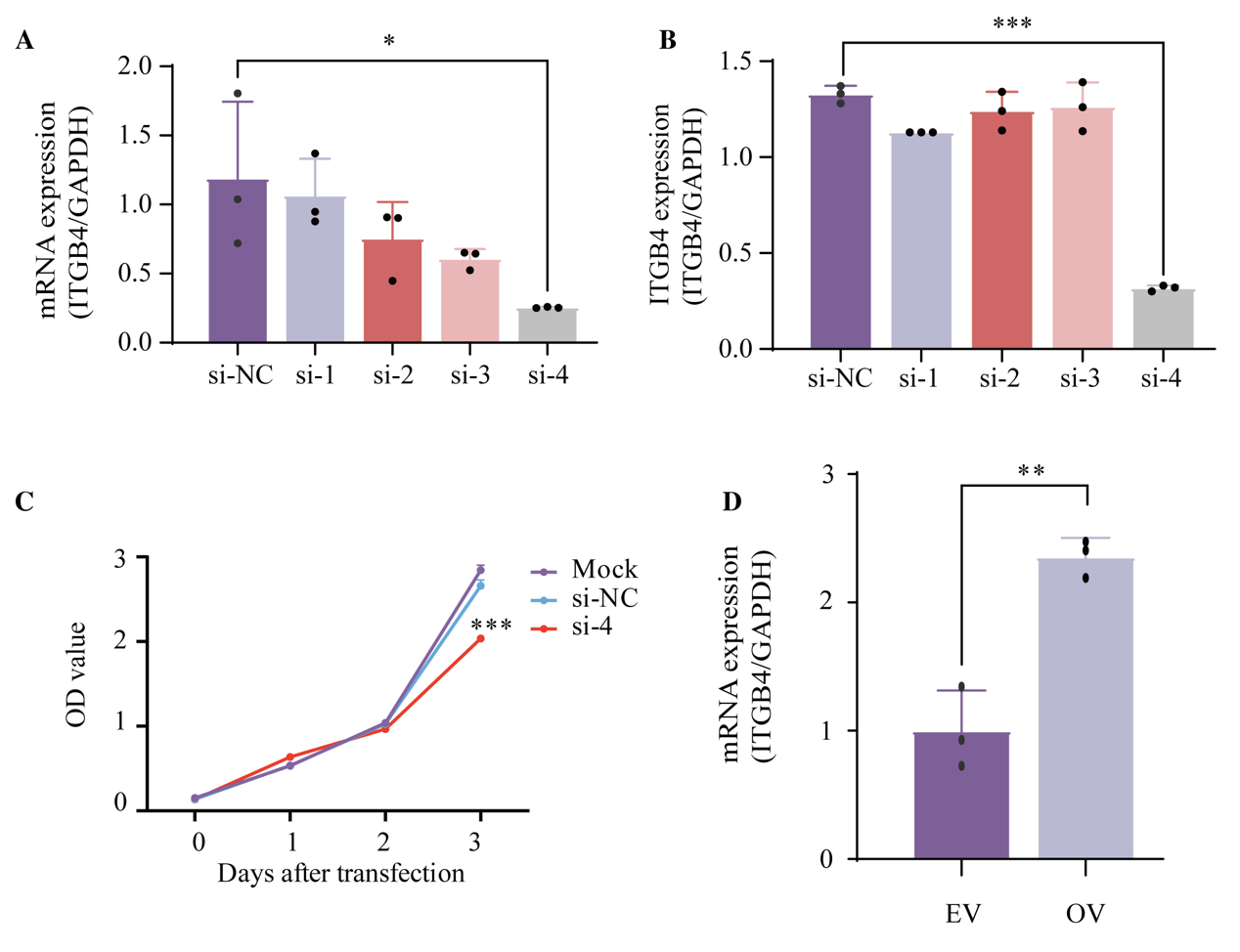


Supplementary Figure 2. ITGB4 is related to proliferation of glioma cells. (A) qPCR and (B) statistical chart of western blot of ITGB4 expression to indicate that we successfully generated U251 cell lines with knocked down ITGB4. (C) Compared to the control cells, U251 cells with knocked-down ITGB4 protein exhibited a significant decrease in cell viability. (D) statistical chart of western blot of ITGB4 expression, indicate that we successfully generated U251 cell lines with over-expressed ITGB4.


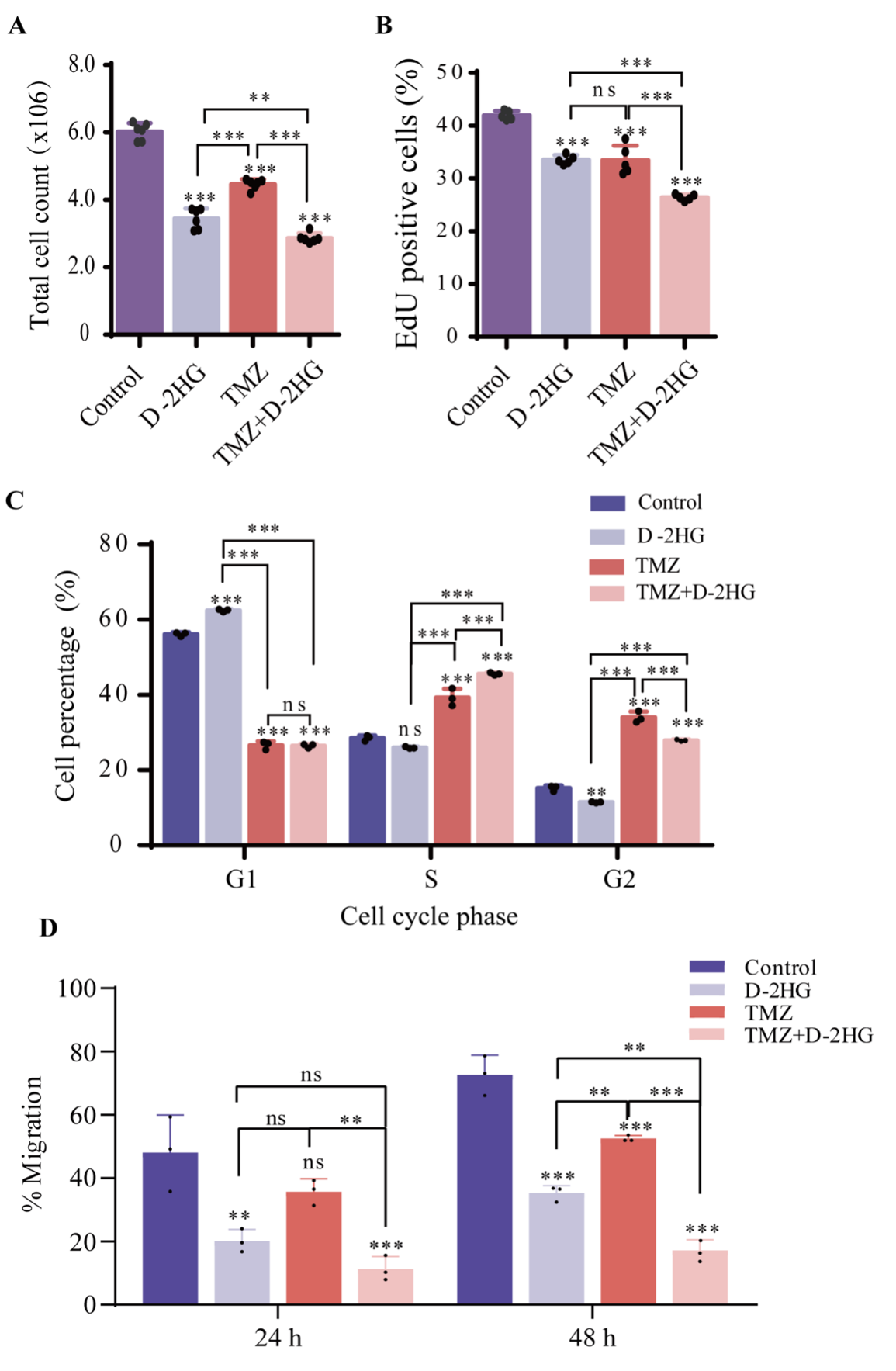


Supplementary Figure 3. D-2HG enhances the anti-proliferative effect of TMZ. U251 cells were treated with D-2HG (500 μM), TMZ (500 μM), or a combination of the two drugs. Percentages of total cell count (A), and EdU positive cell rate (B) were calculated and compared among groups. and percentages of cells in G01, S, and G2 phase (C) and migration rate (D) were calculated and compared among groups.


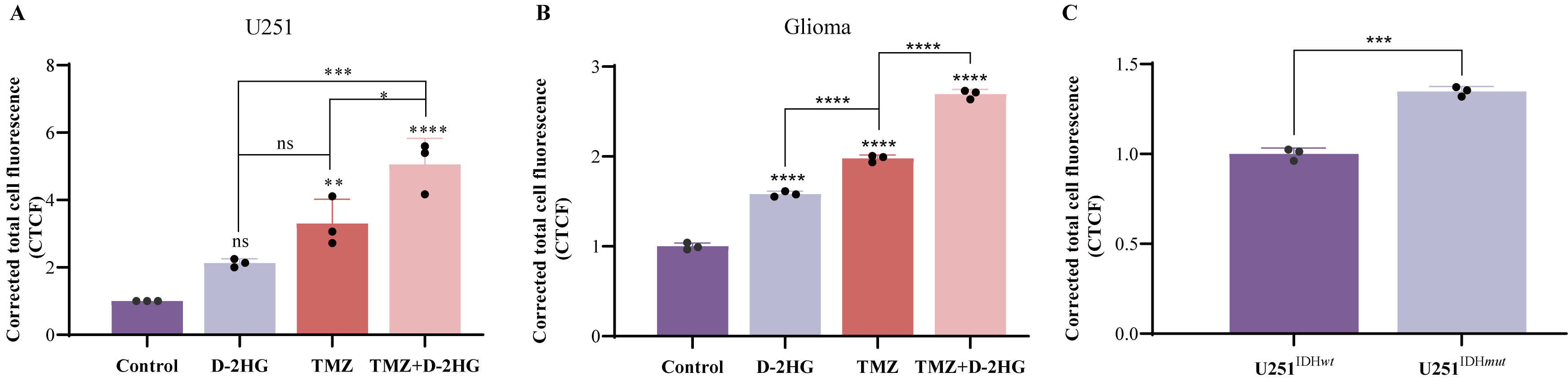


Supplementary Figure 4. Quantification of γH2A.X. (A) γH2A.X levels in U251 cells treated with 500 μM D-2HG, 500 μM TMZ, and 500 μM D-2HG + 500 μM TMZ for 48 hours. (B) γH2A.X levels in glioma cells treated with D-2HG, TMZ, and D-2HG + TMZ for 48 hours. (C) γH2A.X levels in U251^IDH^*^wt^* and U251^IDH^*^mut^* cells after TMZ treatment.

Supplementary Table 1. IDH-mutated gliomas showed elevated D-2HG expression.

| **Case no.** | **Age/Sex** | **Diagnosis** | **WHO** | **IHC** | **D-2HG concentration by HPLC-MS (μM)** |
| --- | --- | --- | --- | --- | --- |
| 1 | 76/M | GBM | Ⅳ | (-) | 24.19 |
| 2 | 41/F | Oligodendroglioma | Ⅲ | (-) | 82.02 |
| 3 | 58/F | GBM | Ⅳ | (-) | 141.85 |
| 4 | 49/F | GBM | Ⅳ | (-) | 133.88 |
| 5 | 59/M | GBM | Ⅳ | (-) | 126.41 |
| 6 | 61/M | Gliosis | / | (-) | 65.37 |
| 7 | 70/M | GBM | Ⅳ | (-) | 24.62 |
| 8 | 70/M | GBM | Ⅳ | (-) | 41.36 |
| 9 | 62/F | GBM | Ⅳ | (-) | 24.45 |
| 10 | 21/M | GBM | Ⅳ | (-) | 44.39 |
| 11 | 45/M | GBM | Ⅳ | (-) | 14.48 |
| 12 | 68/F | GBM | Ⅳ | (-) | 76.99 |
| 13 | 65/M | GBM | Ⅳ | (-) | 27.91 |
| 14 | 70/M | GBM | Ⅳ | (-) | 88.76 |
| 15 | 43/M | GBM | Ⅳ | (-) | 95.17 |
| 16 | 35/M | GBM | Ⅳ | (-) | 97.51 |
| 17 | 69/M | GBM | Ⅳ | (-) | 76.91 |
| 18 | 70/M | GBM | Ⅳ | (-) | 90.36 |
| 19 | 37/F | GBM | Ⅳ | (+) | 944.86 |
| 20 | 31/M | GBM | Ⅳ | (+) | 2652.79 |
| 21 | 51/M | GBM | Ⅳ | (+) | 960.39 |
| 22 | 43/M | Astrocytoma | Ⅳ | (+) | 2425.02 |
| 23 | 44/F | Astrocytoma | Ⅳ | (+) | 284.57 |
| 24 | 47/F | Astrocytoma | Ⅳ | (+) | 1033.80 |
| 25 | 36/F | Astrocytoma | Ⅳ | (+) | 4013.93 |
| 26 | 45/F | Astrocytoma | Ⅲ | (+) | 1855.55 |
| 27 | 47/M | Astrocytoma | Ⅳ | (+) | 4029.90 |
| 28 | 41/M | Astrocytoma | Ⅳ | (+) | 190.74 |
| 29 | 46/M | Astrocytoma | Ⅳ | (+) | 1517.50 |
| 30 | 49/M | Astrocytoma | Ⅳ | (+) | 1098.79 |
| 31 | 30/F | Astrocytoma | Ⅳ | (+) | 2421.90 |
| 32 | 37/M | Astrocytoma | Ⅳ | (+) | 4203.89 |

Supplementary Table 2. primers’ sequence.

| **Primer** | **5’ to 3’** |
| --- | --- |
| ATF2-F | AGGTTCCCAATGTGCTGCTT |
| ATF2-R | GCCTGTTAGAGGATGGTGCC |
| LAMA5-F | GATCGTGGTGTCCCTGGTGA |
| LAMA5-R | GTGTTGGTACGCAGGAAGCG |
| ITGB8-F | CCCCACCTGTTATACAGCCT |
| ITGB8-R | GAGAGCACATGAGGTTTTGC |
| ITGB4-F | CAACAGTGACCCCCCCTTCT |
| ITGB4-R | GGTTGCCTGAGATCCGCTCT |
| CDK2-F | TCTGTTCCAGCTGCTCCAGG |
| CDK2-R | TGATGGCCCCCTCTGTGTTA |
| PI3K-F | AGATCGCTCTGGCCTCATTG |
| PI3K-R | CTTTGTTGAAGGCTGCTGCA |
| AKT-F | TGTGGACCAACGTGAGGCTC |
| AKT-R | AGGCAGCGGATGATGAAGGT |
| hGAPDH-F | agccacatcgctcagacac |
| hGAPDH-R | Gcccaatacgaccaaatcc |
