# Supplementary material for: IDH1-mutant metabolite D-2-hydroxyglutarate inhibits proliferation and sensitizes glioma to temozolomide via down-regulating ITGB4/PI3K/AKT
Source: Cell Death Discov. 2024 Jul 9;10:317. doi: 10.1038/s41420-024-02088-y (PMC11233597; doi:10.1038/s41420-024-02088-y)
Supplement: Supplementary file 2 — Supplementary 1. Chromatographic profiles of D-2HG from the glioma tissues. [file 41420_2024_2088_MOESM2_ESM.docx]

**Supplementary 1. Chromatographic profiles of D-2HG from the glioma tissues.**

**14 IDH1 mutant glioma tissues**

**
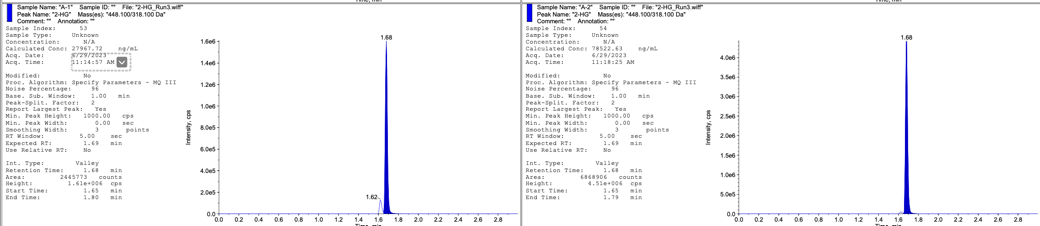

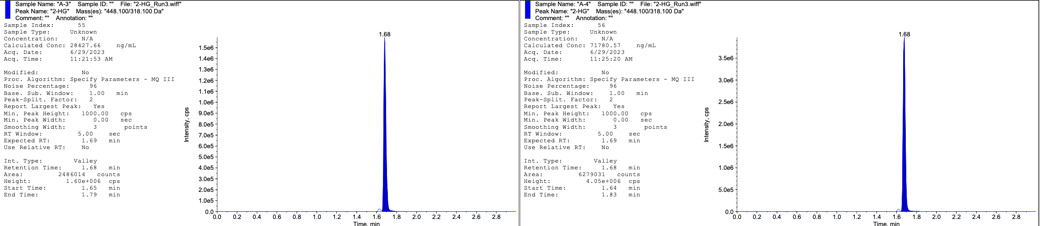
**

**
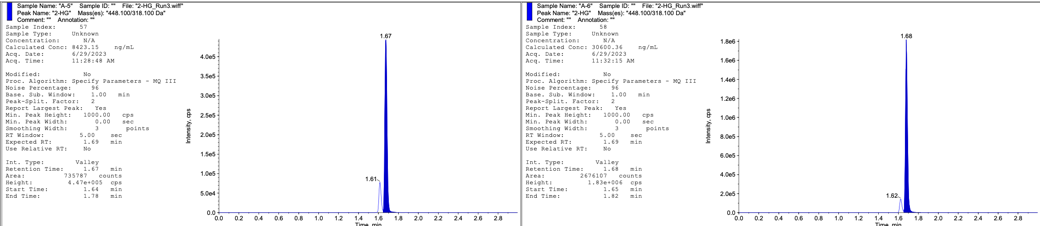

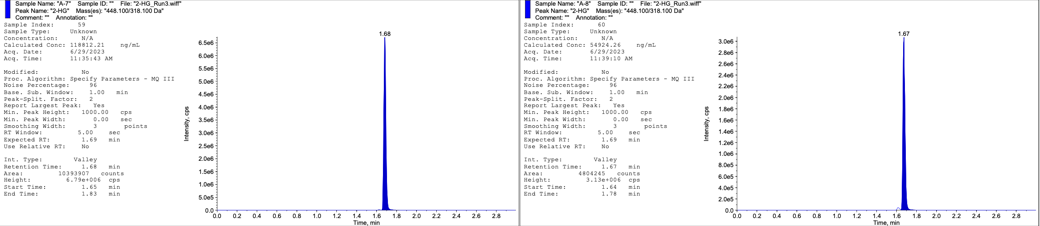
**

**
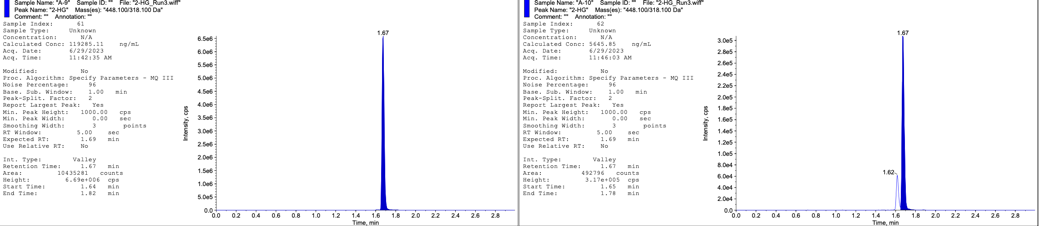

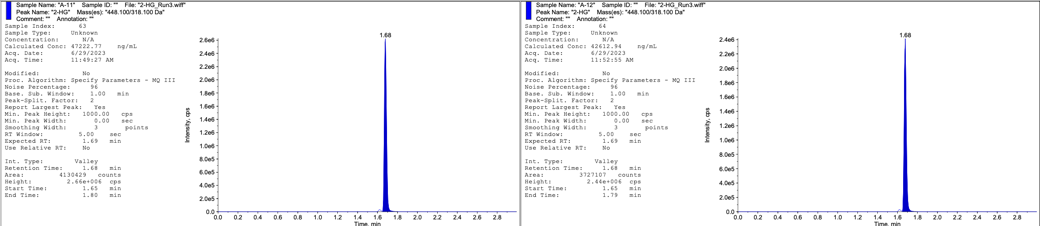
**

**
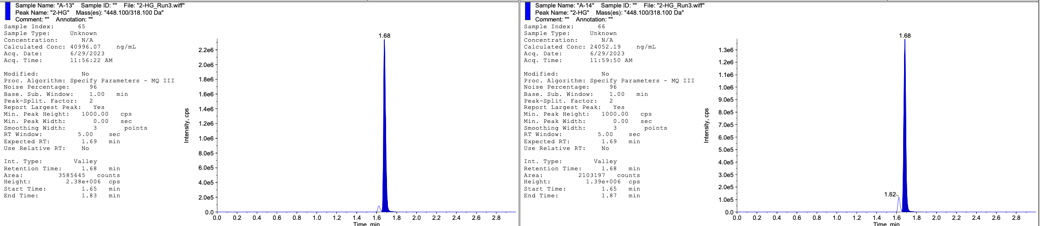
**

**18 IDH1 wild-type glioma tissues**

**
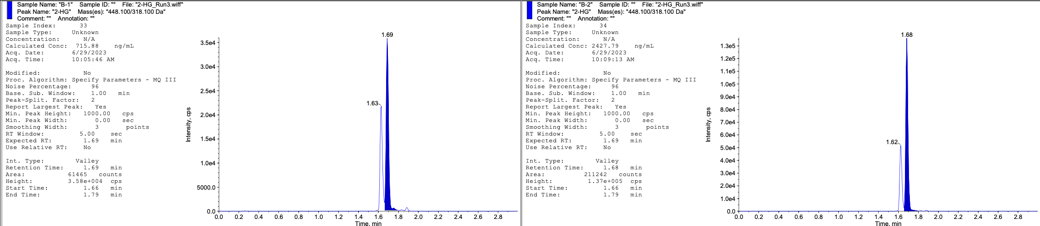

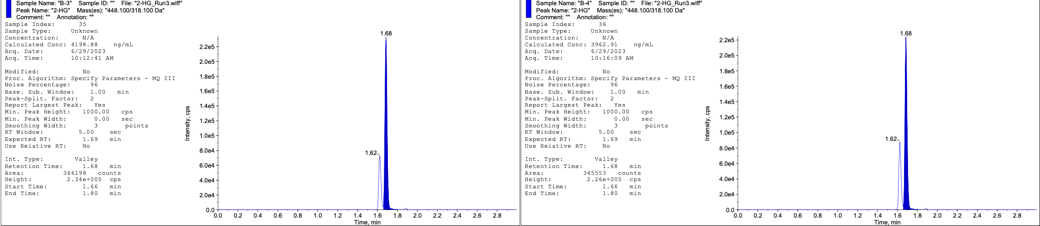

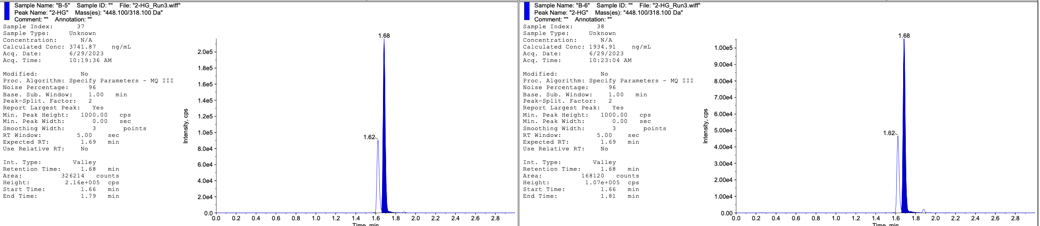

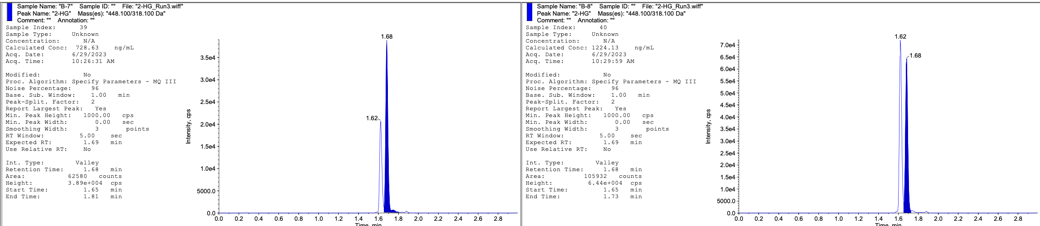

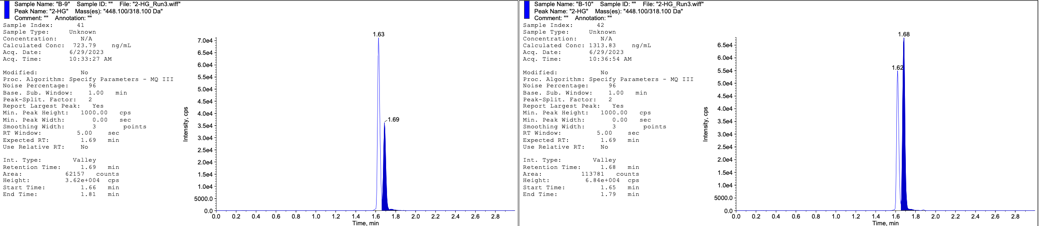

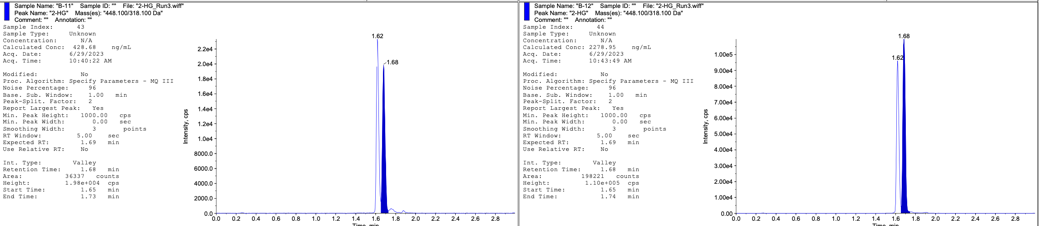
**

**
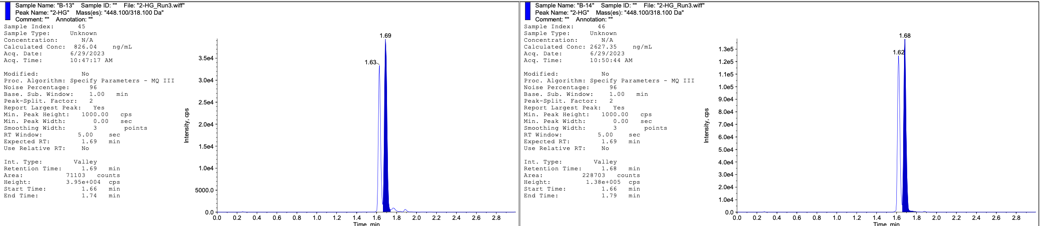

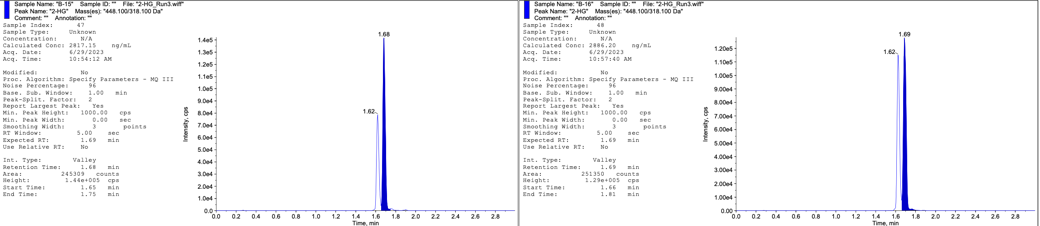
**

**
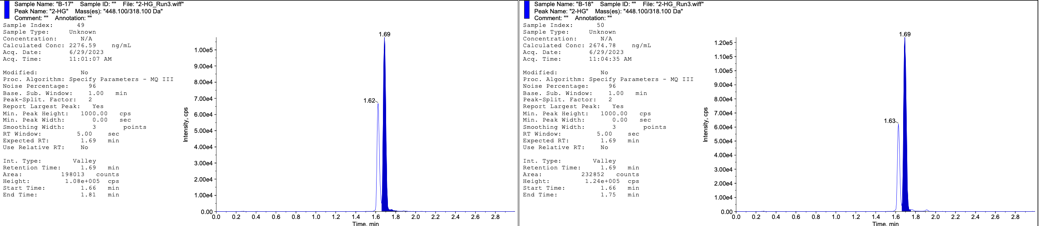
**
