# Supplementary material for: IDH1-mutant metabolite D-2-hydroxyglutarate inhibits proliferation and sensitizes glioma to temozolomide via down-regulating ITGB4/PI3K/AKT
Source: Cell Death Discov. 2024 Jul 9;10:317. doi: 10.1038/s41420-024-02088-y (PMC11233597; doi:10.1038/s41420-024-02088-y)
Supplement: Supplementary file 3 — Supplemental Material-original western blots [file 41420_2024_2088_MOESM3_ESM.pdf]

IDH1-mutant metabolite D-2-Hydroxyglutarate inhibits proliferation and sensitizes glioma to Temozolomide via down-regulating ITGB4/PI3K/AKT

Supplemental Material - the full length uncropped original western blots

Fig 2K.

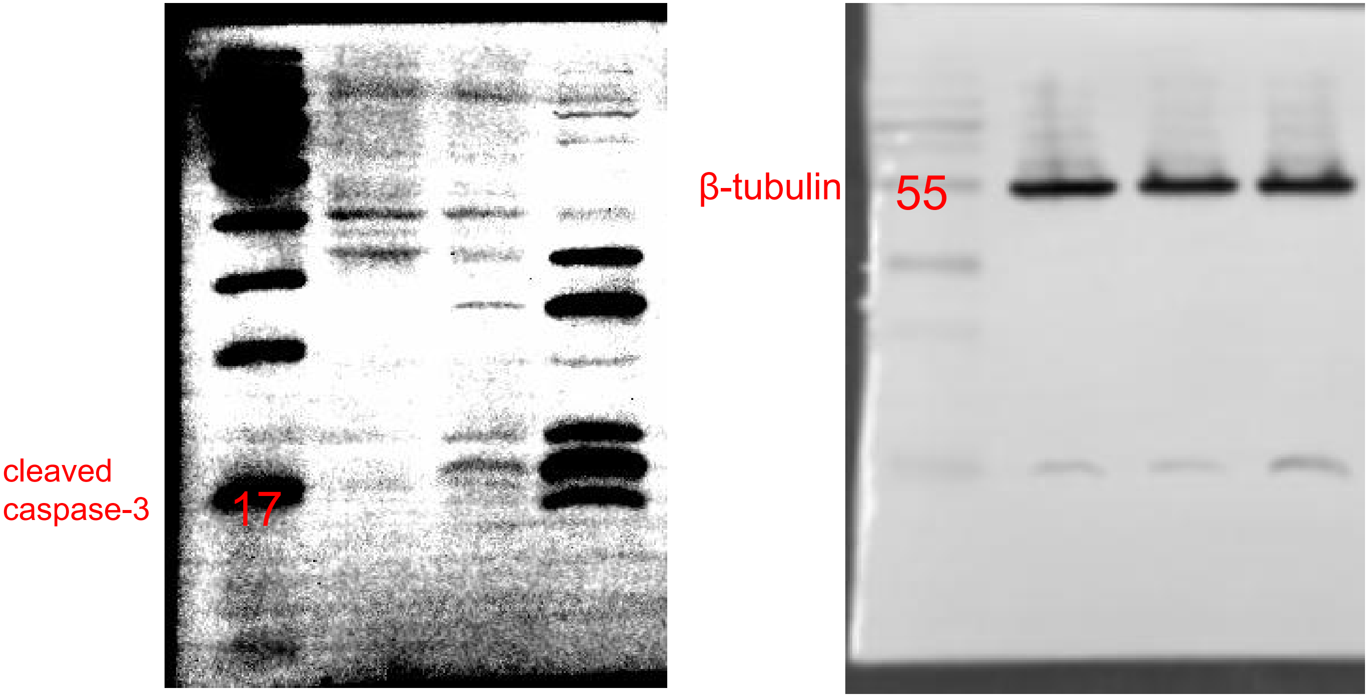

Fig 3E.

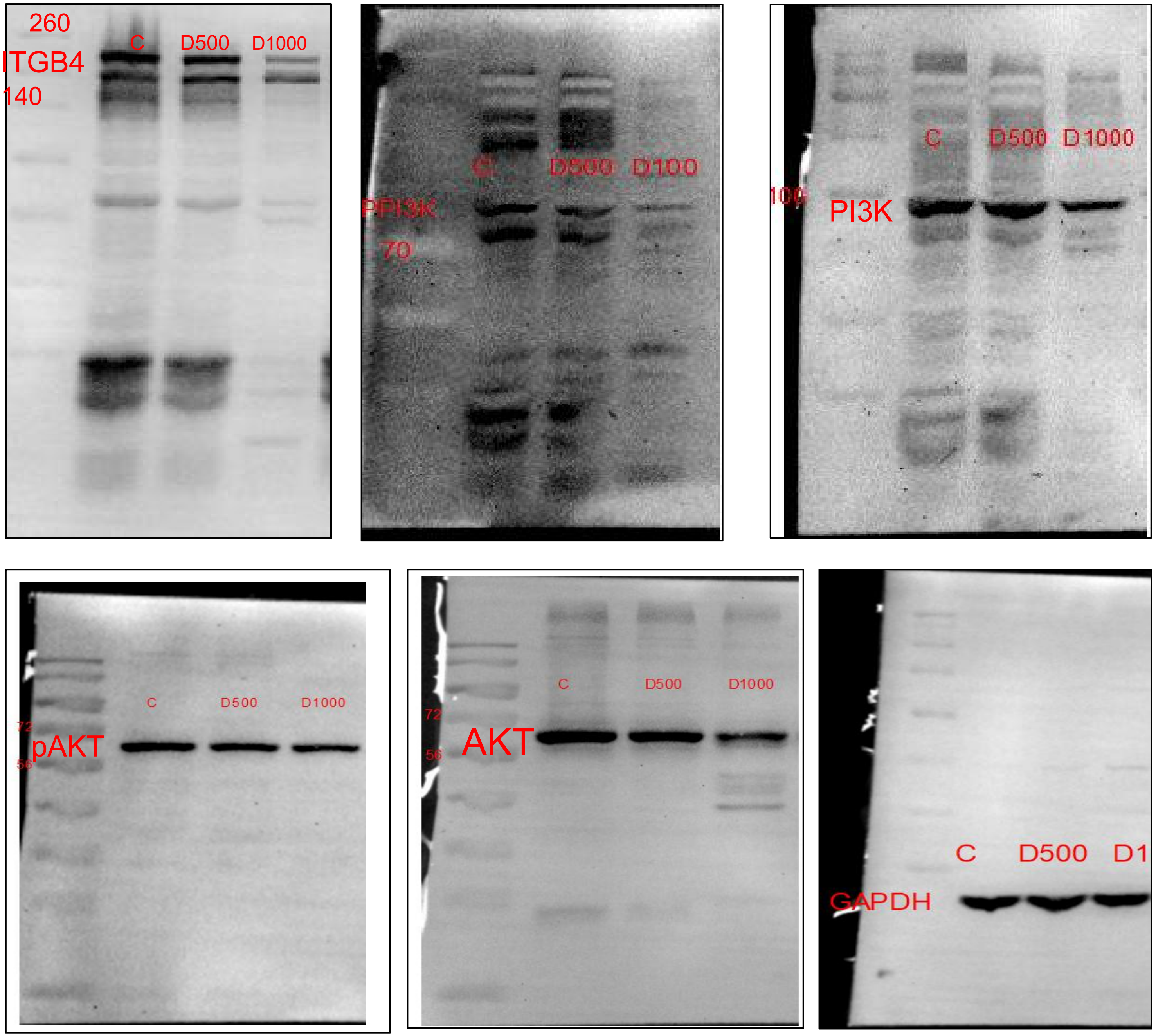

260
ITGB4
140
C
D500
D1000
PPI3K
70
C
D500
D100
100
PI3K
C
D500
D1000
pAKT
72
56
C
D500
D1000
AKT
72
56
C
D500
D1000
GAPDH
C
D500
D1

Fig 4A.

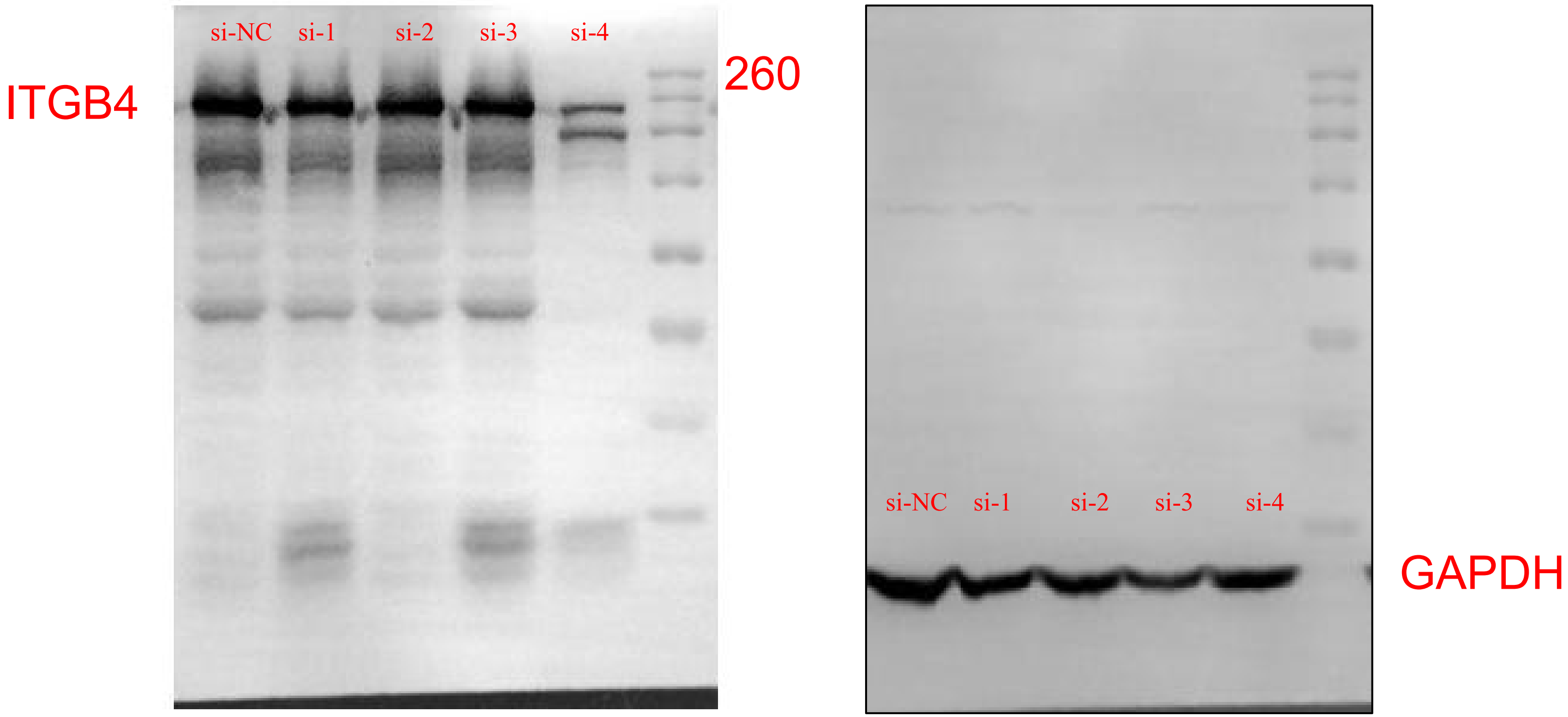

Fig 4G.

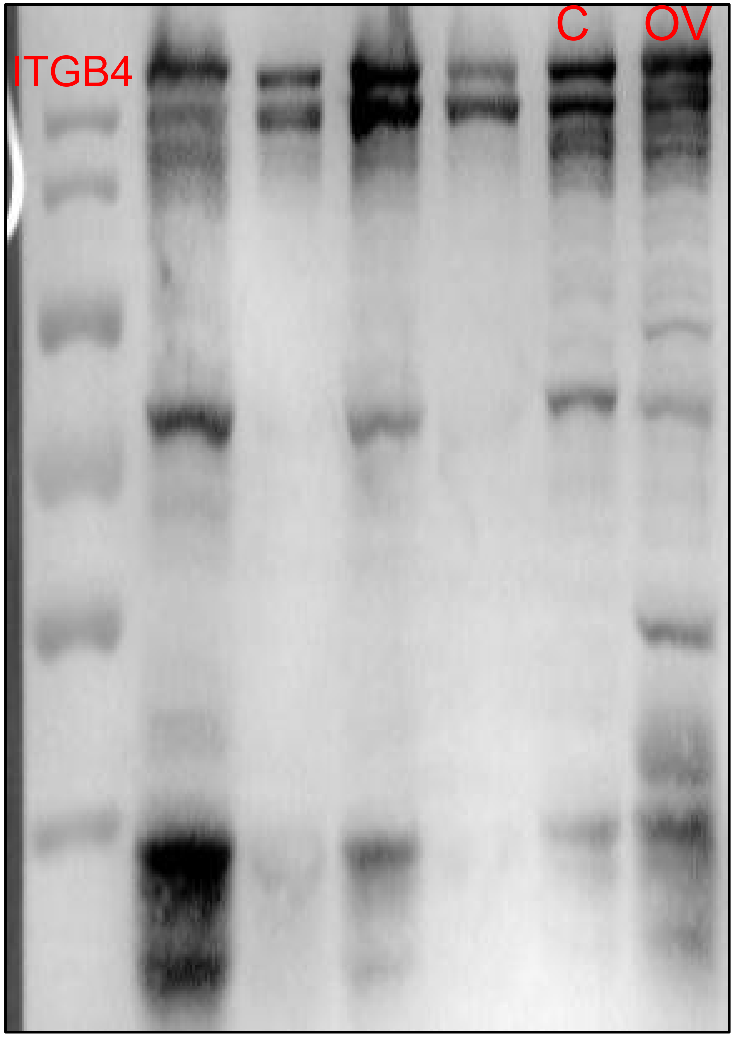

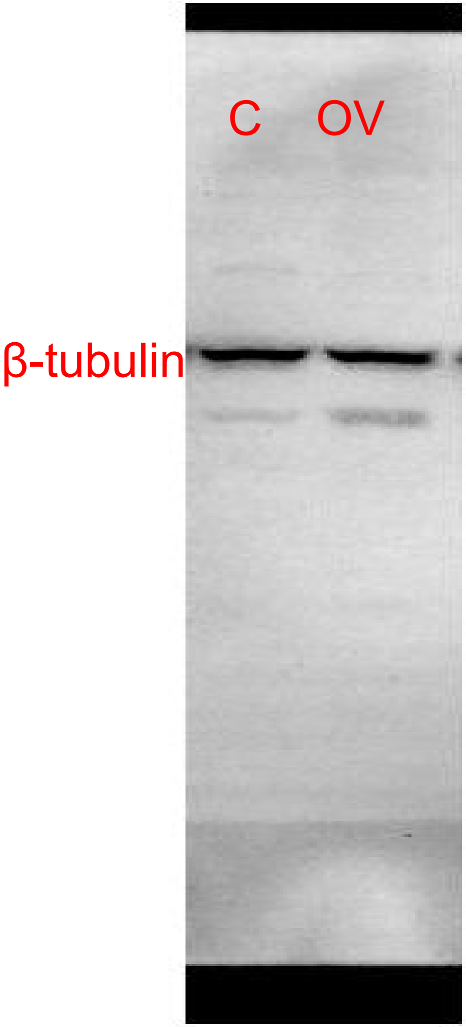

Fig 7A.

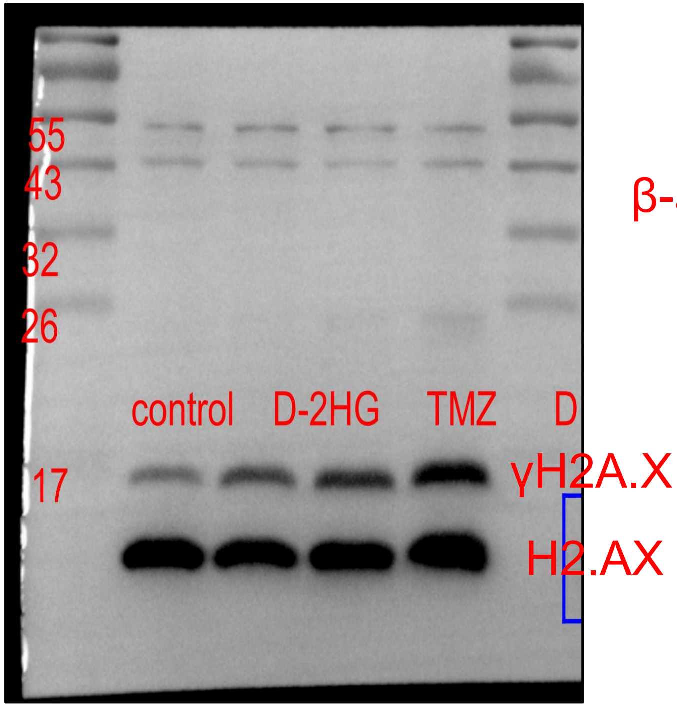
55
43
32
26
17
control
D-2HG
TMZ
D
γH2A.X
H2.AX

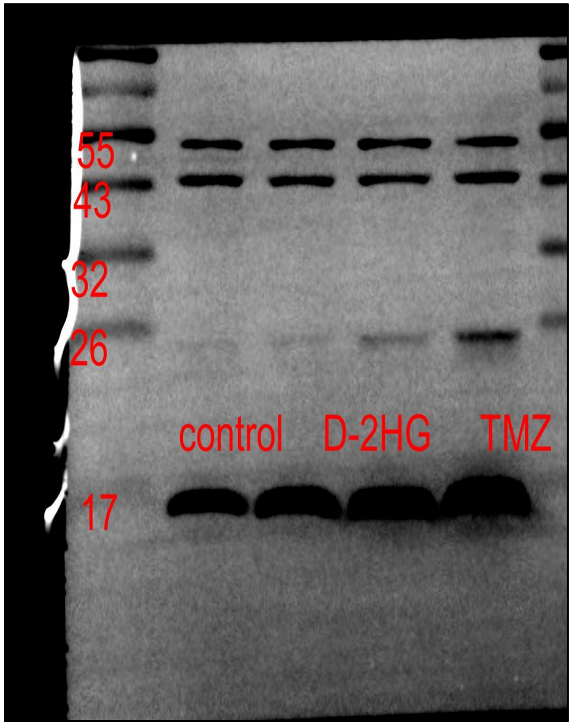
β-actin
55
43
32
26
17
control
D-2HG
TMZ

Fig 8D.

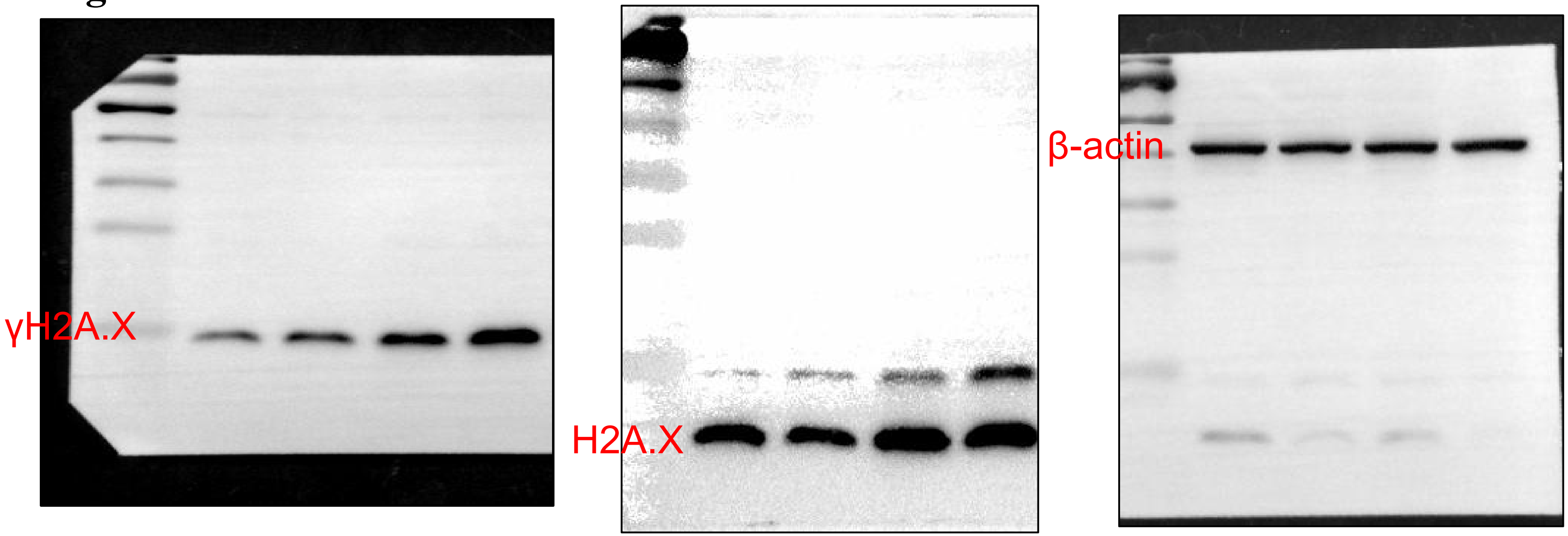

Fig 9A.

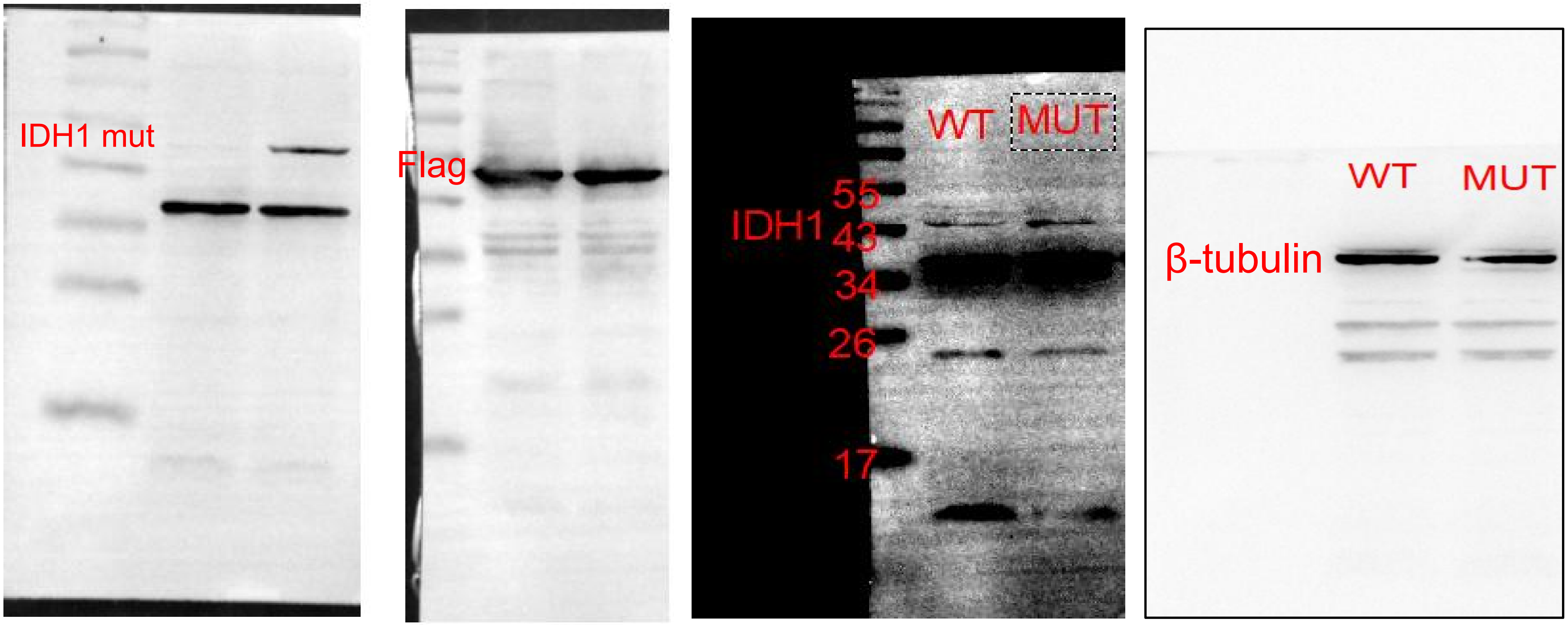

Fig 10A.

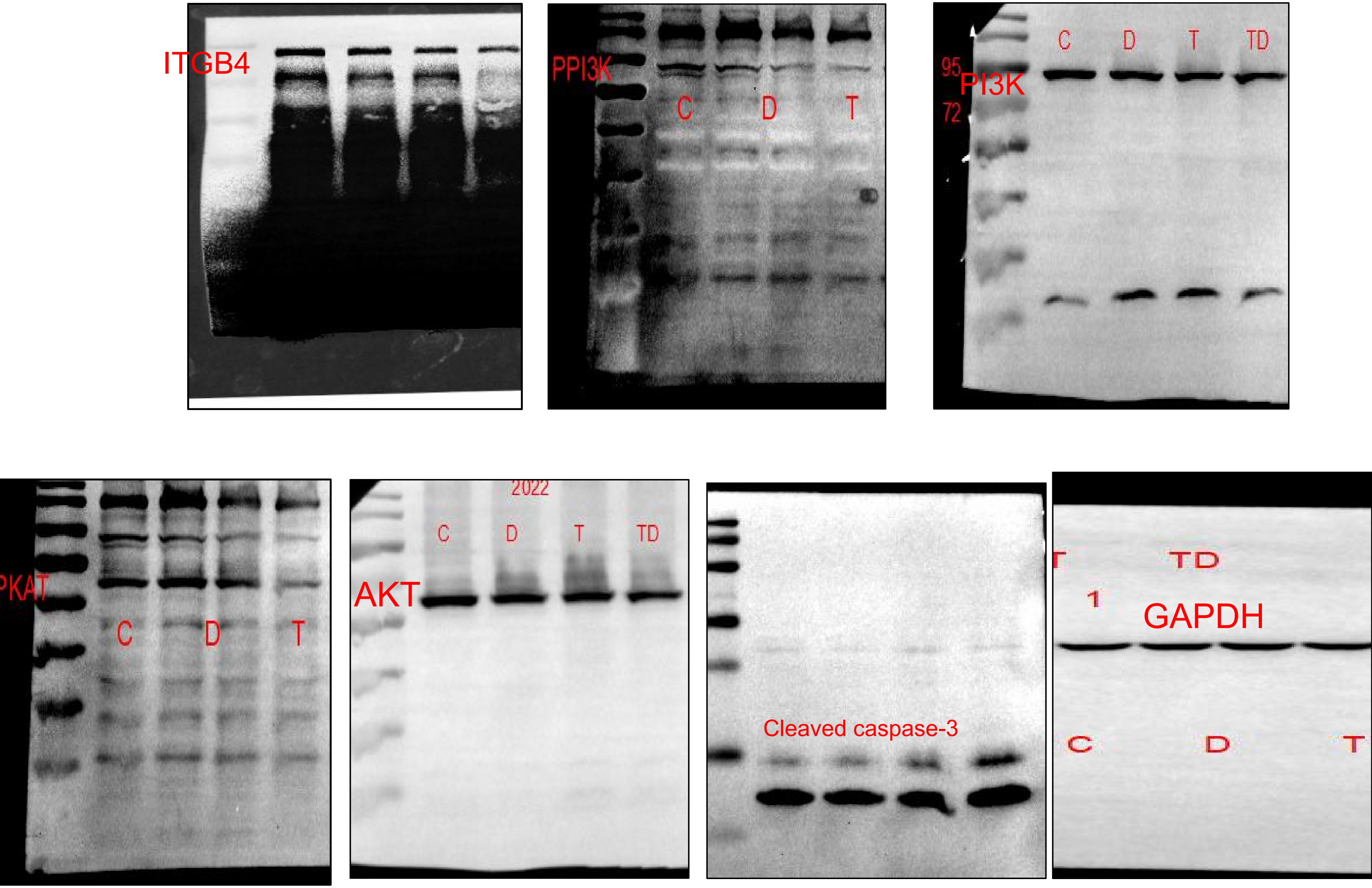
ITGB4
PPI3K
C
D
T
95
72
PI3K
C
D
T
TD
PKAT
C
D
T
2022
AKT
C
D
T
TD
Cleaved caspase-3
T
TD
1
GAPDH
C
D
T
